# Transcriptomic Analysis Reveal the Molecular Mechanisms of Seed Coat Development in *Cucurbita pepo* L.

**DOI:** 10.3389/fpls.2022.772685

**Published:** 2022-02-24

**Authors:** Yingyu Xue, Zhiyan Shen, Fei Tao, Jingjiang Zhou, Bingliang Xu

**Affiliations:** ^1^College of Plant Protection, Gansu Agricultural University, Lanzhou, China; ^2^Biocontrol Engineering Laboratory of Crop Diseases and Pests of Gansu Province, College of Plant Protection, Gansu Agricultural University, Lanzhou, China; ^3^State Key Laboratory Breeding Base of Green Pesticide and Agricultural Bioengineering, Ministry of Education, Guizhou University, Guiyang, China

**Keywords:** seed coat development, transcription profiling, transcription factors, lignin biosynthesis, hulled *Cucurbita pepo*, hull-less *Cucurbita pepo* L.

## Abstract

*Cucurbita pepo* is one of the earliest cultivated crops. It is native to Central and South America and is now widely cultivated all over the world for its rich nutrition, short growth period, and high yield, which make it suitable for intercropping. Hull-less *C. pepo* L. (HLCP) is a rare variant in nature that is easier to consume. Its seed has a seed kernel but lacks a seed coat. The molecular mechanism underlying the lack of seed coat development in the HLCP variety is not clear yet. The BGISEQ-500 sequencing platform was used to sequence 18 cDNA libraries of seed coats from hulled *C. pepo* (CP) and HLCP at three developmental stages (8, 18, and 28 days) post-pollination. We found that lignin accumulation in the seed coat of the HLCP variety was much lower than that of the CP variety. A total of 2,099 DEGs were identified in the CP variety, which were enriched mainly in the phenylpropanoid biosynthesis pathway, amino sugar, and nucleotide sugar metabolism pathways. A total of 1,831 DEGs were identified in the HLCP variety and found to be enriched mainly in the phenylpropanoid biosynthesis and metabolism pathways of starch and sucrose. Among the DEGs, hub proteins (FusA), protein kinases (IRAK4), and several transcription factors related to seed coat development (MYB, bHLH, NAC, AP2/EREBP, WRKY) were upregulated in the CP variety. The relative expression levels of 12 randomly selected DEGs were determined using quantitative real-time PCR analysis and found to be consistent with those obtained using RNA-Seq, with a correlation coefficient of 0.9474. We found that IRAK4 protein kinases, AP2/EREBP, MYB, bHLH, and NAC transcription factors may play important roles in seed coat development, leading to the formation of HLCP.

## Introduction

*Cucurbita* belongs to the family Cucurbitaceae, which originated from the American continent (Paris, 1989; Lin, 2000; Liu et al., 2010). There are five cultivated varieties of *Cucurbita*: *Cucurbita pepo* L., *Cucurbita maxima*, *Cucurbita moschata*, *Cucurbita mixta*, and *Cucurbita ficifolia*, of which *C. pepo* L. had the advantage of strong adaptability and resistance, and often used as a rootstock for *Citrullus lanatus*, *Cucumis sativus* L., and *Cucumis melo*. Hull-less *C. pepo* (HLCP) is a rare type of variation in nature characterized by the lack of a seed coat (Teppner, 2000), which was ate conveniently (Chen et al., 1989; Wang et al., 2001). The hull-less seed coat trait was first discovered in 1982, in a continuously selfed Chinese pumpkin (*C. moschata* Duchesne 65-1-8) cultivar in China (Zhou, 1987). The seeds of HLCP have higher mineral elements (such as Fe, Cu, Ca, Mn, Zn, etc.), unsaturated fatty acid (such as oleic and linoleic acid) and crude fiber content play important role in maintaining normal metabolism and physiological function compared with the seeds of CP *C. pepo*, *C. moschata*, and *C. maxima* (Chen et al., 1989; Wang et al., 2001; Liu et al., 2010). In particular, the Fe content in the seeds of HLCP has been found to be more than 80-times higher than in those of *C. moschata*, which enhance hematopoiesis in humans, and the absence of the same causes diseases such as iron deficiency anemia in the human body (Liu et al., 2010; Ray et al., 2016). Recent study have proved that the effect of anti-proliferative and apoptotic effects on human papillary thyroid carcinoma cell line extracts from hull-less pumpkin (Bahadori et al., 2021). In view of the numerous advantages of HLCP variety, which is convenient to eat and process, and complete kernels can be obtained without shelling. It can also be ate as health oil, with high nutritional value, which is a high-quality source for human body to obtain nutrients.

Studies on the seed coat formation of HLCP *C. pepo* were mainly focused on the phenotypic, physiological and biochemical levels in the early stage, but there was a lack of research on the molecular mechanism of seed coat development. Stuart and Loy (1983) reported that the formation of HLCP variety due to the lack of polysaccharide synthesis and accumulation, rather than a metabolic block in the process of lignin biosynthesis. Loy (2000) found that the seed coat of CP is anatomically composed of epidermis, subcortical, sclerenchyma, soft tissue, and green tissue layers, whereas mutant pumpkin without a seed coat, the cells of sclerenchyma are deformed and disintegrated during seed coat development, and the subcortical and sclerenchyma layers are lost because of the lack of lignin accumulation. However, the research on the molecular mechanism of seed coat development of HLCP *C. pepo* was insufficient and incomplete.

About the studies of seed coat characteristics and genetic mechanisms of CP and HLCP started in the early 1850s (Stuart and Loy, 1983). A large number of orthogonality and backcrossing experiments on CP and HLCP showed that the hulled and hull-less seed coats are controlled by one pair of nuclear genes located at the same site. Hulled is the dominant trait, while hull-less is the recessive trait, which has led many researchers to believe that the hulled and hull-less seed coat are controlled by a pair of quality traits (Zhou, 1987; Li et al., 2011). However, researchers inferred that the development of the seed coat of HLCP was affected by a pair of major genes and some modifying genes, which may be a pair of quantitative traits (Teppner, 2000; Zhang, 2004). Teppner (2000) believed that the seed coat traits of *C. pepo* are controlled by one major effect gene and 6–12 minor effect genes. These studies showed that the genetic mechanism of the seed coat of *C. pepo* is complex and regulated by a pair of major genes and other minor genes.

The lignin synthesis pathway includes phenylpropane, shikimic acid, and specific synthesis pathways, of which were closely related to the formation of the seed coat (Ding et al., 2016). Previous studies have proved that phenylalanine ammonia-lyase (*PAL*), 4-coumarate coenzyme A ligase (*4CL*), and cinnamoyl-coenzyme A reductase (*CCR*) genes were involved in regulation of lignin biosynthesis (Bezold et al., 2005; Chen et al., 2013; Ding et al., 2016). Studies have shown that sucrose synthase (SUS) and plant fructose kinases (FRK) are important enzymes in starch and sucrose metabolic pathways, which were involved in the regulation of lignin biosynthesis (Amor et al., 1995; Doblin et al., 2002; Ruan et al., 2003; Stein et al., 2016). Roach et al. (2012) research found that the FRK plays an important role in the development of xylem fibers in poplar wood, which leads to narrower xylem fibers by inhibiting the expression of cytoplasm FRK2. Chiera and Grabau (2007) proved that the SUS was involved in cell wall biosynthesis of endosperm. Plant seed development is also regulated by transcription factors (TFs) such as MYB, NAC, bHLH, and AP2/EREBP (Kondou et al., 2008; Baud et al., 2016; Huang et al., 2016; Zhu T. et al., 2016; Irish, 2017).

In recent years, RNA Sequencing (RNA-Seq) technology has developed rapidly and been used in many fields (Zhang et al., 2018). However, there is still lack of relevant research on the molecular mechanism of pumpkin seed coat development by transcriptome sequencing. At present, it has been clarified that there are morphological differences in the seed coat development of Hulled and Hull-less *C. pepo* (Xue et al., 2011), but there are no reports on molecular mechanism research. In this study, the developmental mechanism of the seed coats of hulled and hull-less *C. pepo* at three developmental stages (8, 18, and 28 days post-pollination) was revealed by using the transcriptome method (the BGISEQ-500 platform). The results lays the foundation for the optimization of germplasm resources and cultivation of new varieties of hull-less *C. pepo.* Therefore, revealing the formation mechanism of the seed coat of hulled and hull-less *C. pepo* is of great significance for clarifying the seed development of plants.

## Materials and Methods

### Plant Materials and Growth Conditions

Two genotypes of pumpkin materials were used in this study: the inbred line of CP, P3: 03N-123-6 and the inbred line of HLCP, P13: 1N304-7. These were provided by Wuwei Golden Apple Co., Ltd., Wuwei, Gansu, China. The CP and HLCP varieties were planted in the germplasm resources test base of the company. The base has a continental desert climate (He and Pan, 2003), altitude of approximately 1,400 m, average annual precipitation of 110 mm, annual average evaporation of 2,640 mm, and relative humidity of 45%.

Pollination was carried out by means of self-pollination. Pumpkin fruits were collected and their seeds were dissected on the 8th, 18th, and 28th day post-pollination. The surface of the melon was scrubbed with 75% alcohol three times in the laboratory, and the melon seeds were removed and cut into two halves with a blade on an aseptic operation table. The seed kernels were then gently scraped off from the seed coat. The seed kernels and coats were sprayed with small amounts of RNase-OFF™ (Takara Biomedical Technology Co., Ltd., Dalian, China) and DNA-OFF™ (Takara Biomedical Technology Co., Ltd., Dalian, China), and then sterilized with an ultraviolet lamp for 1 h to achieve aseptic and enzyme-free conditions. The seed coats were wrapped in tin foil paper (0.5 g/bag). All samples were frozen in liquid nitrogen immediately after dissection and stored at −80°C until use.

### Determination of Related Enzyme Activities

Lignin content was determined using a semi-quantitative method and measured at OD_540_ (Ju et al., 1993). The activity of *PAL* was determined using the method of Koukol and Conn (1961). The activity of *C*_4_*H* was determined using the method of Lamb and Rubery (1975). The activity of *4CL* was determined using the method of Knobloch and Hahlbrock (1975).

### RNA Extraction, cDNA Library Construction, and Illumina Deep Sequencing

Seed coat samples of the CP and HLCP varieties were collected at 8, 18, and 28 days post-pollination and used for total RNA extraction. Total RNA was extracted separately from the 18 samples (2 varieties × 3 time-points × 3 replicates) using TRIzol™ Reagent (OMEGA Bio-Tek, Guangzhou, China), following the manufacturer’s protocol. The concentration and quality of the total RNA were checked using an Agilent 2100 Bioanalyzer (Agilent Technologies, Waldbronn, Germany). Library construction of mRNAs was performed according to the following protocol: (1) Enrichment and purification of mRNA and polyA tail enriched mRNA using oligo(dT) magnetic beads; The rRNA was hybridized using a DNA probe, to allow for its removal. The DNA/RNA hybrid strand was selectively digested using RNase H, and the DNA probe was removed by digestion with DNase I. (2) The obtained RNA was fragmented using break buffer and reverse transcribed using random N6 primers, which were then synthesized to form double-stranded DNA. (3) The end of the synthesized double-stranded DNA was flattened and phosphorylated at the 5′-end, and the 3′-end formed a sticky end protruding from an “A,” followed by a linker with a protruding “T” at the 3′-end. (4) The ligated products were amplified using PCR with specific primers. The PCR product was thermally denatured into a single strand, and the single-stranded DNA was cyclized using bridge primers to obtain a single-stranded cyclic DNA library. Following cDNA library construction, the cDNA libraries were sequenced using a paired-end (PE) approach with the BGISEQ-500 platform at Wuhan Huada Medical Laboratory Co., Ltd. (Wuhan, China).

### Mapping and Unigenes

To ensure reliability and clean reads, low-quality, pollution, and high joint unknown bases were removed using SOAPnuke (v1.4.0) and Trimmomatic (v0.36). After removing the reads containing linker, the reads with unknown base N content of more than 5%, and low-quality reads with a mass value of less than 15 bases accounted for more than 20% of the total base number of reads.

After obtaining the clean reads, Hisat2 software [v2.1.0, parameters: --dta --phred64 unstranded --new-summary -x index -1 read_r1 -2 read_r2 (PE)]^1^ was used to map and compare the clean reads with the reference genome sequence (Daehwan et al., 2015). The *Cucurbita maxima* genome was used as the reference genome^2^.

The transcripts of each sample were reconstructed using StringTie software (v1.0.4, parameters: -f 0.3 -j 3 -c 5 -g 100 -s 10000 -p 8)^3^, following which the reconstructed information of all samples was integrated using Cuffmerge software (v2.2.1, parameters: -p12)^4^ and compared with the reference annotation information. The transcripts with class code types of ‘‘U,’’ ‘‘I,’’ ‘‘o,’’ and ‘‘J’’ were selected and defined as unigenes. CPC software (v0.9-r2, parameters: default)^5^ was used to predict the protein-coding potential of the unigenes.

### Transcription Factor Identification

GETORF (version: EMBOSS: 6.5.7.0)^6^ software was used to detect the Open Reading Frame (ORF) of the unigenes. Hmmsearch (v3.0, parameters: default)^7^ was used to compare the ORF with the protein domain of TFs. PlantTFDB^8^ was used to identify the ability of unigene to serve as a TF (*E*-value > 10^–5^).

### Identification of Differentially Expressed Genes

The analysis method for DEGs was based on Poisson distribution. DEGseq software (parameter: fold change ≥2 and adjusted *P* ≤ 0.001) was used to detect DEGs based on the MA-plot (Langmead and Salzberg, 2012). To improve the accuracy of DEGs, the *P*-values were calculated according to Normal distribution and then corrected to *Q*-values (Kumar and Futschik, 2007; Wang et al., 2010) with *Q*-value ≤ 0.001 indicating significant differential expression.

### Function Annotation of Differentially Expressed Genes

The DEG sequences were annotated using Gene Ontology (GO; Ashburner et al., 2000) and Kyoto Gene and Genomic Encyclopedia (KEGG; Kanehisa et al., 2004). The DEG sequences were aligned for homology comparison using Blastx, with an *E*-value ≤ 1 × 10^–5^.

### Quantitative Real-Time PCR Analysis

Twelve DEGs were randomly selected from the DEGs for RT-qPCR analysis. The gene-specific primers used in real-time quantitative polymerase chain reaction (RT-qPCR) were designed using Primer Premier 5 software (PREMIER Biosoft, San Francisco, United States) and are listed in Supplementary Table 1. The primer efficiency was checked using melting curve analysis before use. For each sample, 1 μg of total RNA was converted into cDNA using the RevertAid First Strand cDNA Synthesis Kit (Invitrogen, Thermo Fisher Scientific, Waltham, United States). β*-actin* (GenBank accession number: XM_023673479.1) and α*-tubulin* (GenBank accession number: MH310440) were used as internal reference genes (Wu and Cao, 2010). Amplification without a cDNA template served as a negative control. Each amplification reaction was performed using three biological samples/replicates.

RT-qPCR was performed using a Thermo QuantStudio™ 5 thermo cycler (Thermo Fisher Scientific, Waltham, United States). Each reaction (20 μL) consisted of 10 μL TB Green *Premix Ex Taq* II (TIi RNaseH Plus) (TaKaRa, Dalian, China), 0.8 μL forward primer (final concentration 0.4 μM), 0.8 μL reverse primer (final concentration 0.4 μM), 0.4 μL ROX Reference Dye II, 2 μL cDNA template, and 6 μL sterile distilled water. The RT-qPCR program was as follows: 95°C for 30 s, 40 cycles of 95°C for 5 s, and 60°C for 34 s. Relative expression of the selected transcripts was calculated using the 2^–ΔΔCT^ method and REST 2009 software (Qiagen, Hilden, Germany). The efficiency of each pair of primers was tested using melting curve analyses before use (Supplementary Figure 1).

### Comparison of Expression Levels

The Pearson correlation coefficient between RNA-Seq and RT-qPCR expression data was calculated using the cor function in R software (v3.5.1). According to the standard recommended by the Encode program, the square of the Pearson correlation coefficient (R^2^) should be ≥0.92 (ideal sampling and experimental conditions) for two detection techniques that are biologically repetitive (standards, guidelines, and best practices for RNA-Seq).

### Protein-Protein Interaction Network Analysis

Using the STRING protein interaction database^9^, a PPI network was constructed for each group of DEGs, and proteins with a PPI score ≥200 were screened (Liu et al., 2016; Tao et al., 2018). The protein interaction network diagram was constructed using Cytoscape software (v3.6.1).

### Statistical Analysis

Excel 2016 (Microsoft Office 2016) and SPSS 21.0 (IBM SPSS Statistics), were used for data analysis of variance, and REST 2009 software was used to calculate the relative expression of DEGs. One-way ANOVA was used to assess statistically significant differences between the treatments, and Duncan’s new repolarization difference method was used to test the significance of differences among the treatments. Prism 7 (GraphPad) and Illustrator CS6 (Adobe) softwares were used for generating graphs.

### RNA-Seq Data Submission

The raw data for transcriptome assembly and gene expression analysis in this study were submitted to the NCBI Sequence Read Profile (SRA) database, with accession numbers from SRR15439210 to SRR15439227.

## Results

### The Fresh and Dry Weight of Seed Coat Development

**FIGURE 1:**
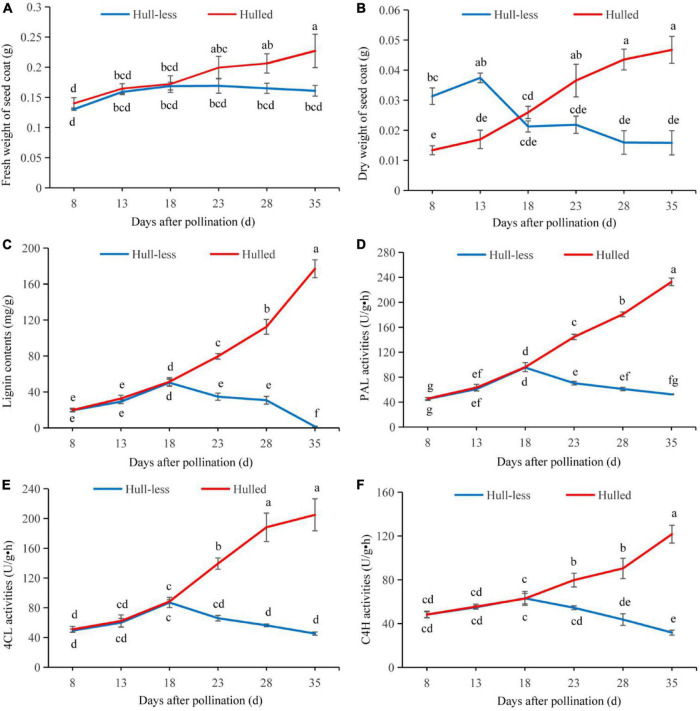
Activities of related enzymes in the seed coat of *C. pepo*, from 8 to 35 days post-pollination (red represents the hulled varieties, while blue represents the hull-less varieties). **(A)** Fresh weight of the seed coat. **(B)** Dry weight of the seed coat. **(C)** Lignin content in the seed coat. **(D)** PAL enzyme activity in the seed coat. **(E)** 4CL enzyme activity in the seed coat. **(F)** C_4_H enzyme activity in the seed coat. For each treatment, the bar represents mean ± SE of three replicates. The different lower case letters represent significant differences at *P < 0.05* levels.

The fresh weights of the seed coats increased from 8 to 18 days post-pollination in both the CP and HLCP varieties, with no significant difference between the groups. At 18–35 days post-pollination, the fresh weight of the CP seed coats continued to increase with the maturity of the seeds, while the fresh weight of the HLCP seed coats showed a downward trend. On the 35th day after pollination, the fresh weight of the CP seed coats was 1.41-times higher than that of the HLCP seed coats (*P* < *0.05*) (Figure 1A).

The dry weight of the HLCP seed coats was higher than that of the CP seed coats from 8 to 18 days post-pollination. The dry weight of the CP seed coats increased gradually from 18 to 35 days post-pollination, whereas that of the HLCP seed coats decreased significantly (*P* < *0.05*) (Figure 1B). On the 35^th^ day post-pollination, the dry weight of the CP seed coats was 2.95-times higher than that of the HLCP seed coats.

### Lignin Content and Related Enzyme Activity

The lignin content and related enzyme activity in the seed coats of the two varieties increased from 8 to 18 days post-pollination, with no significant difference between the two groups. These continued to rise from 18 to 35 days post-pollination in the CP seed coats, but declined in the HLCP seed coats. The lignin content, *PAL*, *4CL*, and *C*_4_*H* enzyme activity in the CP seed coats were 118.68-, 4.44-, 4.52-, and 3.83-times higher in the HLCP seed coats on the 35th day post-pollination than those in the CP seed coats (*P* < *0.05*) (Figures 1C–F).

### Transcriptome of Hulled and Hull-Less *C. pepo*

The RNA samples were suitable for sequencing as the RNA integrity number (RIN) values of all the RNA samples were between 7.3 and 9.1 (Supplementary Table 2). The samples were named according to the variety, sampling days post-pollination, and number of biological replicates (Supplementary Figure 2); for example, CP81 referred to the sample of the hulled variety (CP) collected at 8 days post-pollination, and number 1 among three replicates. The average correlation coefficient of all the 18 samples was 0.95 (Supplementary Figure 3), minimum correlation coefficient was 0.9108 (between CP81 and CP83), and maximum correlation coefficient was 0.9857 (between HLCP181 and HLCP182) (Supplementary Table 4).

Sequencing using the BGISEQ-500 platform produced an average yield of 10.88 Gb per sample and a total of 196 Gb data with a Q30 of more than 89.19%. A total of 209,453 million raw reads and 195,759 clean reads were produced (Supplementary Table 3). A total of 18,332 new transcripts or unigenes were detected, of which 16,958 were known protein-coding transcripts, 356 belonged to unknown protein-coding transcripts, and the remaining 1,018 belonged to non-coding transcripts (Supplementary Table 7). The transcript length distributions of the known genes are shown in Supplementary Figure 4. Among these transcripts, 3,378 (9%) were less than 500 nt, 6,086 (17%) were between 500 and 1,000 nt, 7,775 (21%) were between 1,000 and 1,500 nt, 6,787 (19%) were between 1,500 and 2,000 nt, 4,513 (12%) were between 2,000 and 2,500 nt, 2,829 (8%) were between 2,500 and 3,000 nt, and 4,750 (13%) were longer than 3,000 nt.

Among these unigenes, the group with 7,775 (21%) transcripts accounted for the largest proportion, with sizes between 1,000 and 1,500 nt, and the group with 2,829 (8%) transcripts accounted for the least proportion, with sizes between 2,500 and 3,000 nt (Supplementary Figure 4A).

### Identification of Differentially Expressed Genes

**FIGURE 2:**
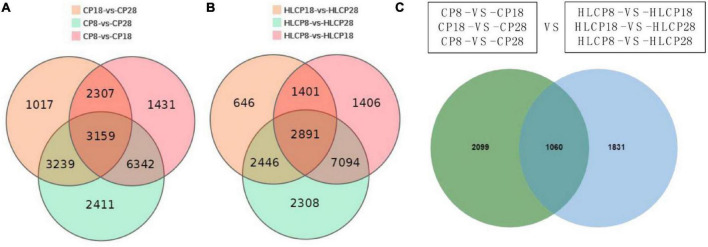
Venn diagram of differentially expression genes (DEGs). **(A)** DEGs of hulled variety. **(B)** DEGs of hull-less variety. **(C)** Venn diagram showing the number of unique DEGs in the two varieties.

A Venn diagram has been constructed to show the differential genes in different varieties at different growth stages (Figure 2). In total, the expression of 3,159 DEGs was found in CP samples [(CP8 *vs.* CP18) *vs.* (CP18 *vs.* CP28) *vs.* (CP8 *vs.* CP28) comparisons] (Figure 2A). The expression of 2,891 DEGs was found in HLCP samples [(HLCP8 *vs.* HLCP18) *vs.* (HLCP18 *vs.* HLCP28) *vs.* (HLCP8 *vs.* HLCP28) comparisons] (Figure 2B). Among the 6,050 DEGs, 1,060 were identified in both CP [(CP8 *vs.* CP18) *vs.* (CP18 *vs.* CP28) *vs.* (CP8 *vs.* CP28) comparisons] *vs.* HLCP [(HLCP8 *vs.* HLCP18) *vs.* (HLCP18 *vs.* HLCP28) *vs.* (HLCP8 *vs.* HLCP28) comparisons] comparisons. Thus, there were 3,930 (2,099 + 1,831) unique DEGs, of which 2,099 and 1,831 were expressed in the CP and HLCP varieties, respectively (Figure 2C).

### Functional Annotation and Enrichment Analysis of Differentially Expressed Genes

There were 3,159 (2,099 + 1,060) DEGs in the CP variety and 2,891 (1,060 + 1,831) DEGs in the HLCP variety, of which 2,099 and 1,831 DEGs were unique in the CP and HLCP varieties, respectively (Figure 2C). Among the 2,099 DEGs in the CP variety, upon KEGG analysis, 142 were found to be significantly enriched in phenylpropanoid biosynthesis (ko00940), phenylalanine metabolism (ko00360), amino sugar and nucleotide sugar metabolism (ko00520), and diterpenoid biosynthesis (ko00904) (*Q*-value < 0.05, Table 1). Notably, 60 DEGs and 23 DEGs were enriched in phenylpropanoid biosynthesis and phenylalanine metabolism, respectively. Among the 1,831 DEGs belonging to the HLCP variety, upon KEGG analysis, 118 were significantly enriched (*Q*-value < 0.05, Table 1) in starch and sucrose metabolism (ko00500), phenylpropanoid biosynthesis (ko00940), cutin, suberin, and wax biosynthesis (ko00073), and brassinosteroid biosynthesis (ko00905).

**TABLE 1:** Significant KEGG pathways enrichment analysis of DEGs.

Type	Pathway name	DEGs with pathway annotation	All genes with pathway annotation	*P*-value	*Q*-value	Pathway ID
	Phenylpropanoid biosynthesis	60 (13.48%)	445 (2.76%)	3.08E-11	3.79E-09	ko00940
Hulled	Amino sugar and nucleotide sugar metabolism	44 (10.35%)	425 (2.64%)	2.05E-05	0.000630203	ko00520
*C. pepo*	Phenylalanine metabolism	23 (15.03%)	153 (0.95%)	6.56E-06	0.000268944	ko00360
	Diterpenoid biosynthesis	15 (12.50%)	120 (0.74%)	0.001827797	0.04496381	ko00904

	Starch and sucrose metabolism	54 (8.50%)	635 (3.94%)	6.66E-05	0.00439501	ko00500
Hull-less	Phenylpropanoid biosynthesis	46 (10.34%)	445 (2.76%)	1.71E-06	0.000225827	ko00940
*C. pepo*	Cutin, suberin and wax biosynthesis	11 (17.74%)	62 (0.38%)	0.000199236	0.006574781	ko00073
	Brassinosteroid biosynthesis	7 (19.44%)	36 (0.22%)	0.001650619	0.043576342	ko00905

*The values of correct-p (Q-value) <0.05 are considered.*

Comparison of the Fragments Per Kilobase per Million (FPKM) values between the CP and HLCP transcriptomes revealed that the expression levels of some unigenes were significantly higher in the CP variety than in the HLCP variety, at 28th day post-pollination. These included 4-coumarate-CoA ligase 1 (CmaCh01G002000.1), cytochrome P450 protein (CmaCh07G004450), heme-binding peroxidase (CmaCh02G012170), cinnamyl alcohol dehydrogenase 1 (CmaCh04G004700), beta-glucosidase (CmaCh10G009490), and transposon Ty2-C Gag-Pol polyprotein (CmaCh11G019740), the expression levels of which were 41.63-, 3.29-, 77.71-, 55.91-, 63.50-, and 25.50-times higher, respectively, in the CP variety than that in the HLCP variety.

**FIGURE 3:**
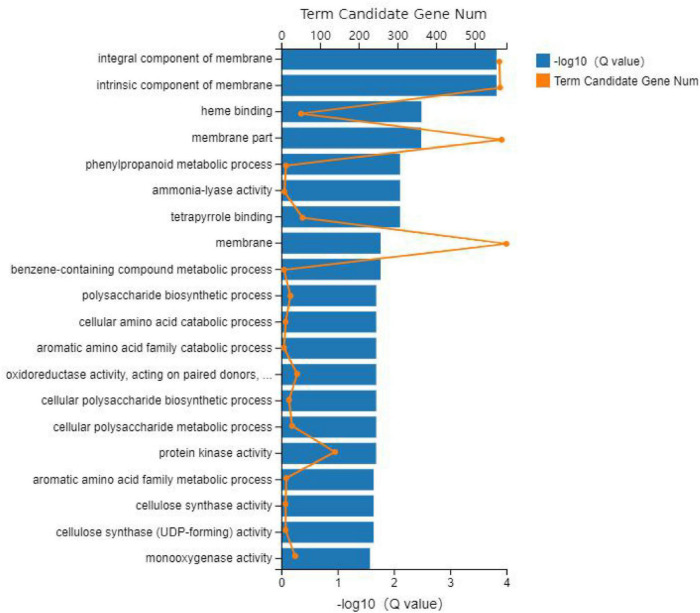
GO enrichment figure of 2,099 DEGs in the seed coat of hulled *C. pepo*.

**FIGURE 4:**
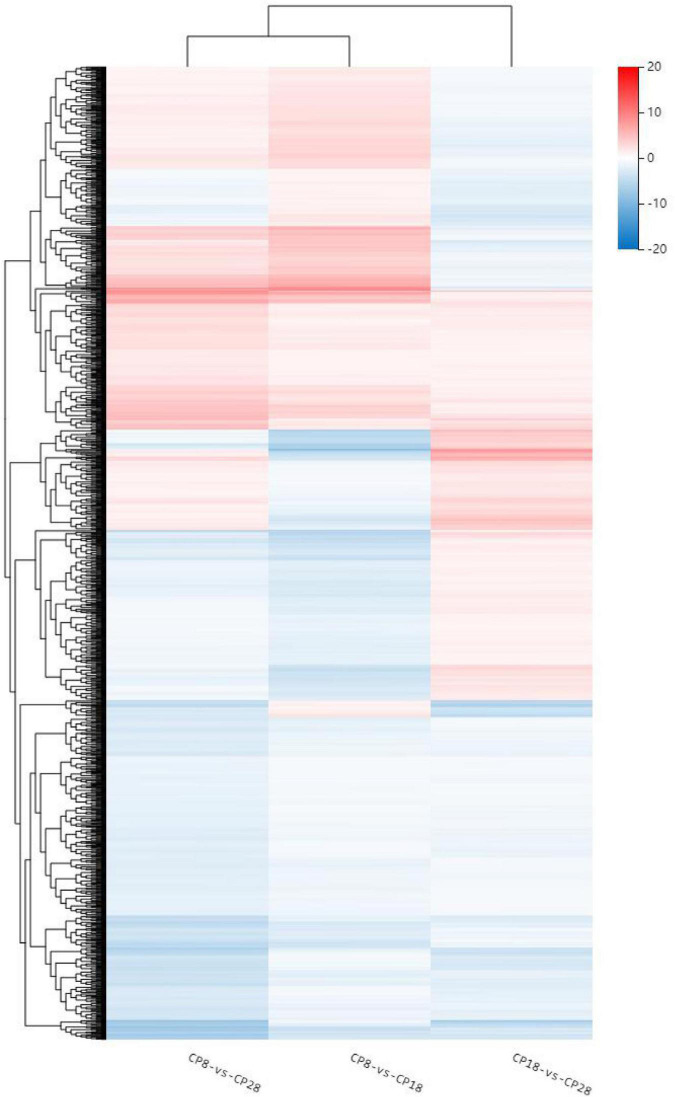
Heatmap of GO cluster of 2,099 DEGs in the seed coat of hulled *C. pepo*.

**FIGURE 5:**
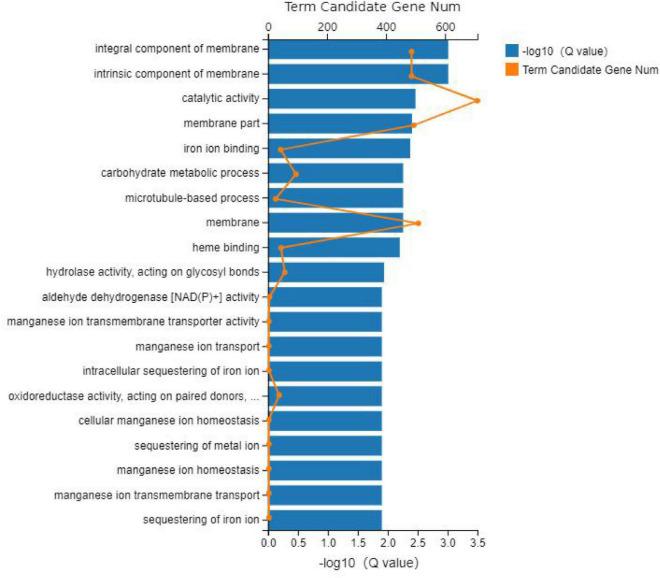
GO enrichment figure of 1,831 DEGs in the seed coat of hull-less *C. pepo* L.

**FIGURE 6:**
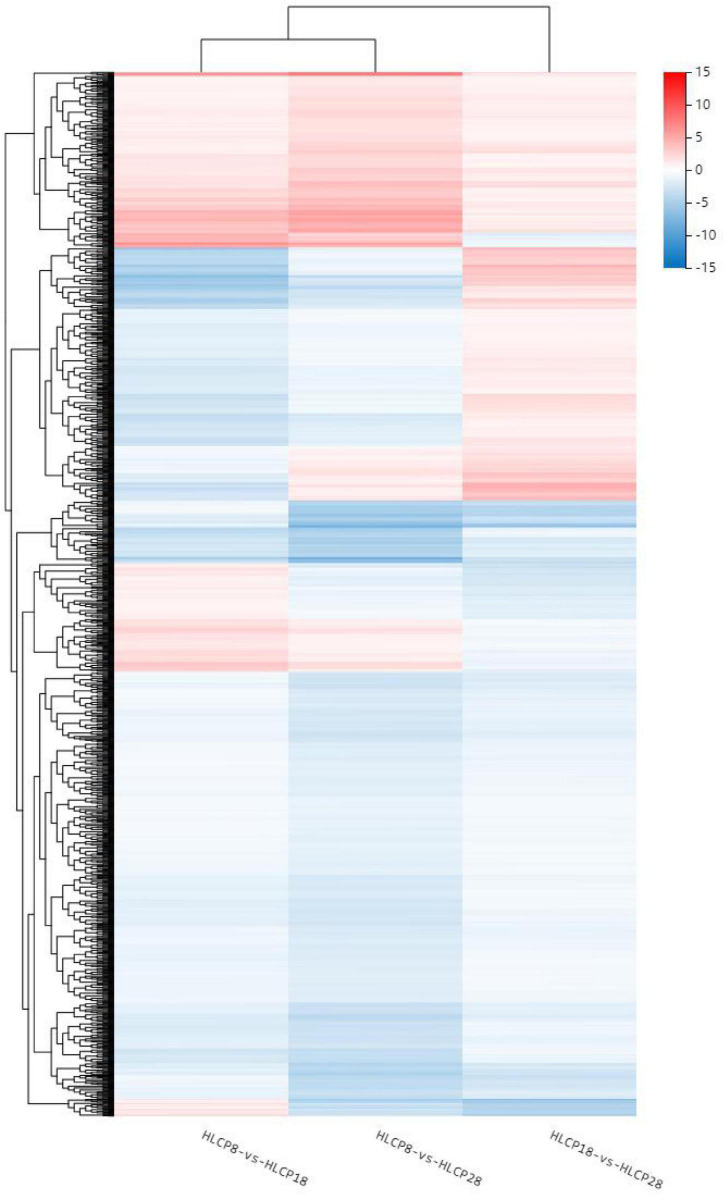
Heatmap of GO cluster of 1,831 DEGs in the seed coat of hull-less *C. pepo* L.

The GO database was used to enrich and classify the 2,099 DEGs of the CP variety and 1,831 DEGs of the HLCP variety. The DEGs of CP were enriched in 21, 4, and 14 terms of biological process categories, cellular components, and molecular functions, respectively (*Q*-value < *0.05*) (Figures 3, 4). The DEGs of HLCP were enriched in 11, 4, and 19 terms of biological process categories, cellular components, and molecular functions, respectively (Figures 5, 6). GO enrichment results showed that the 2,099 DEGs of CP variety and 1,831 DEGs of HLCP variety were mainly involved in the following four common pathways: integral component of membrane, intrinsic component of membrane, membrane part, and membrane (Supplementary Tables 8, 9). In addition, the 2,099 DEGs of the CP variety included genes involved in phenylpropanoid biosynthetic process, PAL activity, and cellulose synthase activity (Supplementary Table 8). The 1,831 DEGs of the HLCP variety included genes involved in catalytic activity, transferase activity, and protein kinase activity (Supplementary Table 9).

### Starch and Sucrose Metabolism Involved in Seed Coat Development

Among the 1,831 DEGs in the HLCP variety, 54 genes were significantly enriched in starch and sucrose metabolism (ko00500) based on KEGG analysis (Supplementary Data 1). Among them, 23 genes were upregulated in the seed coat of CP variety after 28 days of pollination. In particular, sucrose synthase gene (CmaCh10G007940), trehalose 6-phosphate phosphatase gene (CmaCh16G005260), the fructokinase gene (CmaCh17G006030), glucan endo-1,3-beta-glucosidase gene (CmaCh09G006850), and endoglucanase gene (cmach04g008740), the expression levels of which were 9.48-, 24.17-; 3.51-, 18.78-; 0.99-, 7.49-; 8.82-, 7.11-; and 3.65-, 7.09- fold times, respectively, in the CP variety higher than those in the HLCP variety after 18 days and 28 days of pollination. According to the FPKM value of the above genes and the Fold change differential expression in the seed coat of CP and HLCP varieties, it can be considered that these genes were closely related to the formation of seed coat.

### Transcription Factors Involved in Seed Coat Development

**FIGURE 7:**
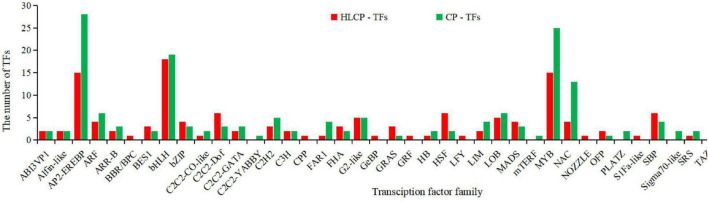
Analysis of transcription factors of DEGs between the seed coats of hulled and hull-less *C. pepo.*

In this study, 187 DEGs from the 2,099 unique DEGs in the CP variety were identified in 37 TFs families (AP2/EREBP, bHLH, MYB, and NAC). Among the unique 1,831 DEGs in the HLCP variety, 149 DEGs were identified as 39 TFs (AP2/EREBP, bHLH, MYB, and WRKY) (Supplementary Data 2 and Figure 7).

Among these TFs, AP2-EREBP (AP2 domain), Myb_DNA-binding domain (MYB), NAM domain (NAC), WRKY domain (WRKY), and bHLH are the families with the largest number of genes. Moreover, the number of genes in the HLCP variety was significantly lesser than that in the CP variety, with numbers of 15 *vs.* 28, 15 *vs.* 25, 4 *vs.* 13, and 8 *vs.* 12, respectively. Eighteen TFs were upregulated in the CP variety and were considered to be related to its seed coat formation (Table 2). Among them, the expression level of the eukaryotic translation initiation factor 2C (ELF2C) gene (BGI_novel_G000014) of the ABI3VP1 TF family was upregulated 41.68- and 146-times in the seed coat of the CP variety, as compared to that of the HLCP variety, at 18 days and 28 days post-pollination, while the amidase [EC: 3.5.1.4] gene (BGI_novel_G000306) of the AP2-EREBP TF family was upregulated 86.70- and179-times, the CYP92A6 (Typhasterol/6-deoxotyphasterol 2 alpha-hydroxylase) gene (CmaCh01G000290) of the bHLH TF family was upregulated 662.67- and 224.29-times, and the *4CL* gene (CmaCh01G002000.1) of the C2C2-GATA TF family was upregulated 35.54- and 41.63-times (Supplementary Data 2 and Supplementary Figure 5).

**TABLE 2:** Transcription factor analysis of 2,099 DEGs in hulled *C. pepo*.

Name of TFs	Gene Num	Up-range
ABI3VP1	1	146.00
Alfin-like	1	2.32
AP2-EREBP	6	3.33–179.00
ARF	1	1.95
bHLH	3	3.93–224.29
C2C2-CO-like	1	9.75
C2C2-Dof	1	127.00
C2C2-GATA	1	41.63
C2C2-YABBY	1	3.48
FHA	1	5.85
GRAS	1	34.57
LIM	1	3.86
LOB	1	4.02
MYB	14	1.33–18.67
NAC	6	1.03–2.19
Sigma70-like	1	2.52
TCP	1	4.01
WRKY	7	2.06–9.31
Total: 18	49	

Among the 149 genes related to the HLCP variety, 39 genes (23 TFs) were upregulated and considered to be related to the seed coat formation of the HLCP variety (Table 3). In particular, the expression level of the polyadenylate-binding protein (*PABPC*) gene (CmaCh01G011940) of the MYB TF family was upregulated 3.61- and 140.16-times in the seed coat of the CP variety at 18 days and 28 days post-pollination, as compared with that of the HLCP variety, while transforming growth factor-beta-induced protein (*TGFBI*) gene (CmaCh01G005390) of the G2-like TF family was upregulated 628.14- and 111.76-times, auxin-responsive protein IAA (*IAA*) gene (CmaCh01G000940) of the bZIP TF family was upregulated 5.01- and 17.78-times, and uridine kinase (*Udk*) gene (BGI_novel_G000078) of the Alfin-like TF family was upregulated 12.49- and 16.37-times. According to the FPKM values of the above genes and the differential expression folds in different varieties, it can be considered that these genes and their TF families are closely related to the formation of seed coat (Supplementary Data 2 and Supplementary Figure 5).

**TABLE 3:** Transcription factor analysis of 1,831 DEGs in hull-less *C. pepo* L.

Name of TFs	Gene Num	Up-range
ABI3VP1	2	1.40–1.46
Alfin-like	2	2.37–16.37
AP2-EREBP	4	1.30–8.84
ARF	1	1.65
ARR-B	1	4.99
BES1	1	7.52
bHLH	4	4.81–25.05
bZIP	2	3.13–17.78
C2C2-Dof	1	6.98
C2H2	1	3.22
C3H	1	2.14
CPP	1	2.09
G2-like	1	111.76
GRAS	1	3.70
HB	1	2.75
LIM	1	5.54
LOB	1	3.24
MYB	9	1.71–140.16
NAC	1	2.16
SBP	3	3.12–8.77
SRS	1	2.36
Trihelix	1	2.20
WRKY	2	1.78–3.00
Total: 23	39	

### Protein Interaction Network Analysis of Related Proteins Involved in Seed Coat Development in Hulled and Hull-Less *C. pepo*

**FIGURE 8:**
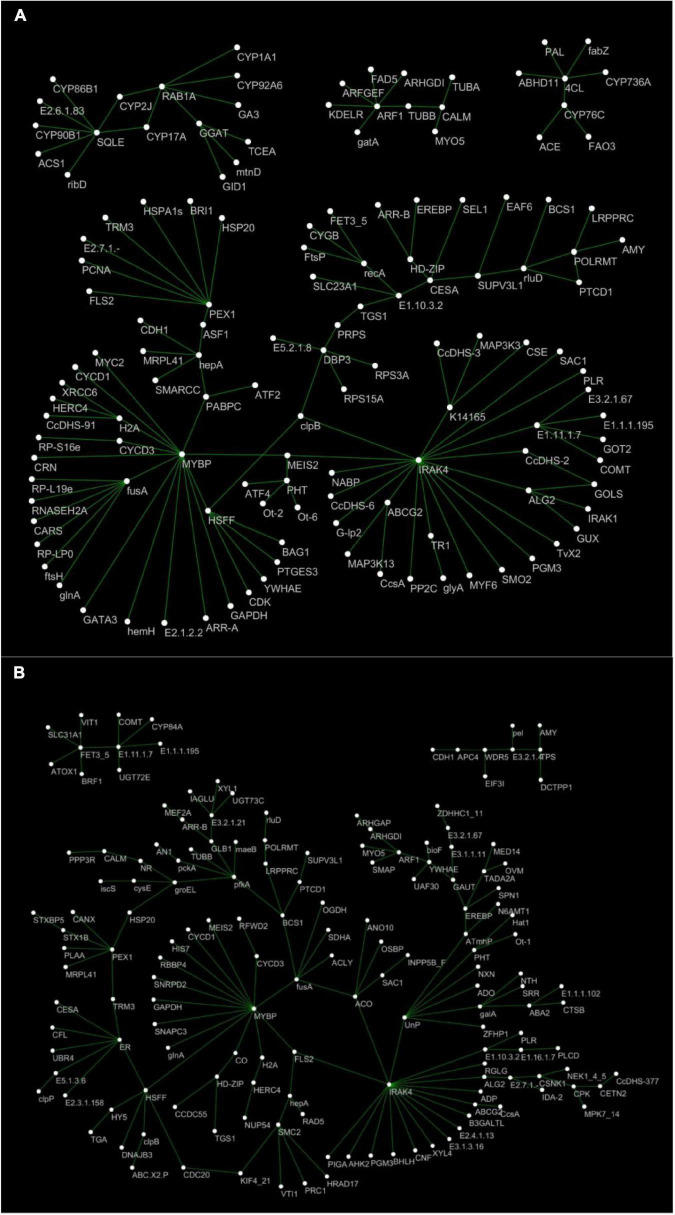
Protein interaction network in the seed coat of hulled and hull-less *C. pepo.* Each node represents a DEG. **(A)** PPI network of 1,831 DEGs in the seed coat of hull-less *C. pepo* L. **(B)** PPI network of 2,099 DEGs in the seed coat of hulled *C. pepo*.

Of the unique 2,099 DEGs in the CP variety, protein interaction network analysis identified 494 proteins with a PPI score of >200. These proteins were associated with the development of seed coat of the CP variety (Supplementary Data 3 and Figure 8A), and mainly included interleukin-1 receptor-associated kinase 4 (IRAK4), clpB, PEX1 protein, HSFF, and MYB TFs. MYB TFs and IRAK4 are hub proteins that interact with 14 and 18 proteins, respectively. For example, MEIS2 interacts with IRAK4 and MYB TFs. MYB TFs interact with IRAK4 protein kinase in two ways: MYB-FLS2-IRAK4 and MYB-fusA-ACO-IRAK4. In addition, proteins related to lignin biosynthesis, such as *4CL* and PAL, form a separate interaction network and are upregulated in the CP variety.

Similarly, 415 proteins were identified from the unique 1,831 DEGs in the HLCP variety (Supplementary Data 3 and Figure 8B), which mainly consisted of proteins such as IRAK4, fusA, HSFF, fls2, pfkA, ACO, and MYB TFs. Compared to the CP variety, most MYB TFs were downregulated in the HLCP variety. In addition, ACO, fusA, and pfkA are hub proteins that interact with 5, 6, and 21 proteins, respectively.

### Verification of RNA-Seq Analysis Using RT-qPCR

**FIGURE 9:**
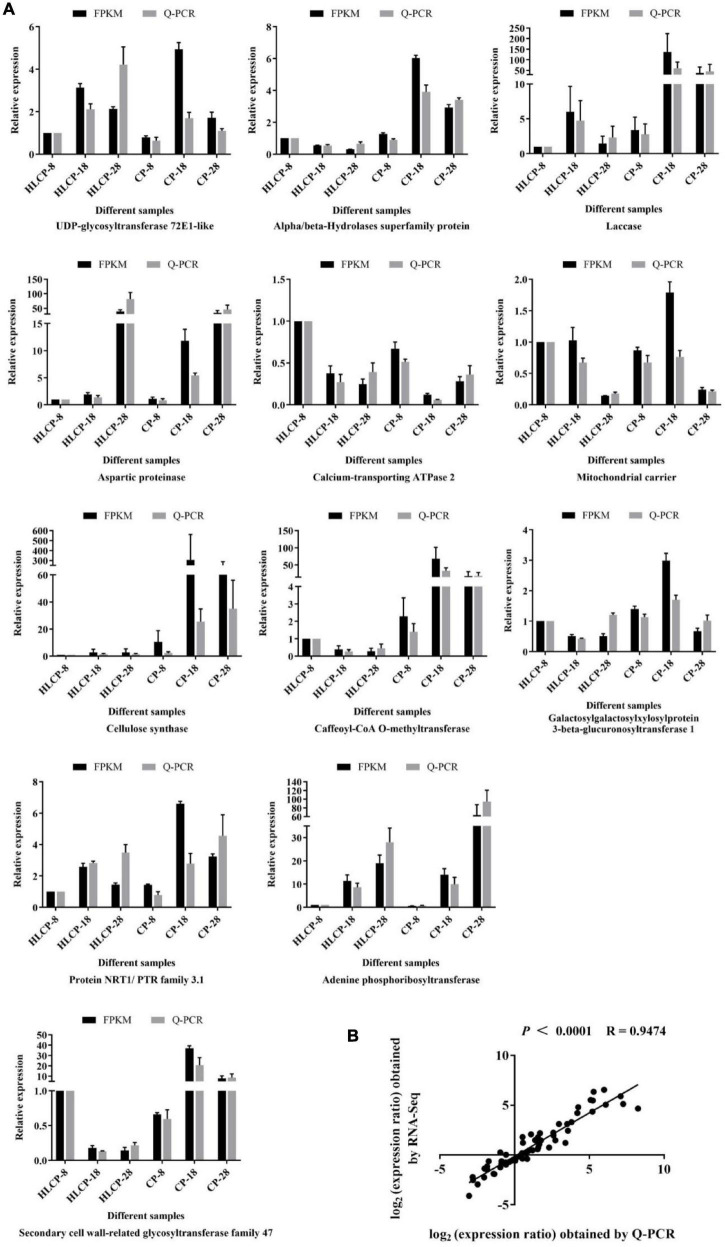
Verification of transcriptome analysis of 12 genes using qRT-PCR. **(A)** The relative expression levels and RNA-Seq FPKM levels of 12 randomly selected transcripts were verified using qRT-PCR. The black bar indicates the relative expression level of RNA-Seq, which is the FPKM value. The relative gene expression of genes analyzed using qRT-PCR is represented by means of a gray histogram. Error bars represent standard error calculated from three replicates. **(B)** Correlation analysis of log_2_ value between RNA-Seq and qRT-PCR values.

Twelve transcripts were randomly selected from the DEGs and analyzed using RT-qPCR to verify the gene expression profile. HLCP-8 samples were used as controls to calculate the relative expression of genes in the CP variety. Figure 9 shows that the relative expression levels obtained using RNA-Seq analyses were consistent with those of RT-qPCR (Figure 9A), with a correlation coefficient of 0.9474 (*P* < 0.0001; Figure 9B).

## Discussion

### Identification of Differentially Expressed Genes Related to Seed Coat Formation in Hulled and Hull-Less *C. pepo*

Understanding the molecular mechanism of seed coat formation of HLCP is of great significance for the breeding of HLCP varieties. Previous studies have reported the inheritance and seed coat structure in CP and HLCP. These studies showed that the content of lignin and cellulose in the seed coat was the main determinant of seed hardness. Lignin is a natural component of the secondary cell wall that participates in the formation of thick-walled cells and structural fibers (Dalimov et al., 2003). Liang et al. (2006) also found similar findings in a study on *Arabidopsis* seed coats. Abnormal lignin synthesis or metabolism in hull-less seeds may lead to a decrease in lignin accumulation in the seed coat cell wall. Some lignin biosynthesis genes have been cloned and analyzed from the perspective of transcriptional regulation (Zhong and Ye, 2009; Zhou et al., 2009; Weng and Chapple, 2010). Similarly, transcriptome sequencing of the seeds of soft-seed and hard-seed pomegranate revealed differential gene expression between soft and hard pomegranate varieties and identified high expression of lignin-related genes and cellulose synthesis-related genes in hard pomegranates. In soft-seed pomegranate, flavonoids and programmed cell death-related genes are slightly higher (Xue et al., 2017). Using high-throughput RNA-Seq technology, 2,099 and 1,831 DEGs were identified to be involved in the seed coat development of CP and HLCP, respectively. In addition, the expression levels of DEGs in the CP and HLCP varieties were consistent with their developmental histological data. In this study, the genes related to seed coat formation, which include the TF gene, protein kinase, and genes of unknown function, were identified based on the mapping assembly strategy using RNA-Seq. Based on morphological observations of seed coat development and analysis of DEGs in transcriptome data, we speculated that the lack of hypodermis and sclerenchyma layers in the seed coat of hull-less varieties may be related to the regulation of some DEGs, TFs, protein kinases, and genes related to lignin biosynthesis.

### Synthetic Process of Lignin Are Positively Involved in Seed Coat Formation

Lignin biosynthesis was closely related to the development of seed coat. The decreasement of lignin accumulation leads to seed coat degradation (Shi et al., 2019). Lignin synthesis was mainly involved in three pathways, including phenylpropanoid metabolic pathway, shikimic acid pathway and lignin synthesis pathway. *PAL*, *4CL*, and *CCR* are the key enzymes in the phenylpropanoid metabolic pathway. Dai et al. (2013) reported that the DEGs were mainly enriched in lignin biosynthesis pathway in soft-endocarp and hard-endocarp hawthorn, and most genes related to lignin pathway (such as *PAL*, *4CL*, and *CCR*) were downregulated in soft-endocarp hawthorns. Xue et al. (2017) found that the DEGs in soft-seed and hard-seed pomegranate were mainly enriched in lignin biosynthesis and metabolic pathways, and most genes related to lignin synthesis (such as *C4H* and *CCR* genes) and cellulose synthesis were upregulated in hard-seed pomegranate. Yu et al. (2019) showed that the DEGs of the hardening period of walnut peel were significantly enriched in phenylpropanoid biosynthesis, phenylalanine metabolism and other pathways by using the Gene Ontology and the KEGG databases.

In this study, KEGG enrichment results of DEGs showed that a large number of DEGs were enriched in phenylpropanoid biosynthesis and phenylalanine metabolism. In particular, the expression levels of *PAL* (CmaCh07G009140) and *4CL* (CmaCh01G002000.1) genes in the CP variety on day 18 days and 28 days after pollination were 32.63-, 35.54- and 214.00-, 41.63-times, which were higher than those in the HLCP variety, respectively. This results were consistent with the previous studies. It proved that lignin biosynthesis and metabolic pathways were closely related to seed coat formation.

### Starch and Sucrose Metabolism Are Positively Involved in Seed Coat Formation

Fructose kinases (FRKs) and sucrose synthase (SUS) were involved in starch and sucrose metabolic pathways, which play an important role in the growth and development of plant seeds. Damari-Weissler et al. (2009) proved that the *SlFRK2* gene in tomato is essential for proper vascular development as stems of SlFRK2-antisense plants have xylem vessels and xylem fibers with thinner secondary cell walls and the xylem vessels of those plants are also narrower and deformed. The tomato plastidic FRK, SlFRK3 is also important for xylem development. The combined suppression of both the *SlFRK2* in cytosolic and the *SlFRK3* in plastidic disturbed not only xylem vessels, but also the development of xylem fibers, resulting in unlignified and distorted xylem fibers (Stein et al., 2016). German et al. (2003) reported that the *LeFRK2* gene can influence the accumulation of fructose and sucrose, and regulated the development of cell wall and vascular in tomato stem, indicating that the FRKs plays an important role in secondary cell wall synthesis. Stein et al. (2017) showed that the *AtFRKs* genes play a key role in carbon metabolism in embryos during seed maturation and the accumulation of storage reserves in *A. thaliana*. Although the early stage of seed development is related to cell division and embryo morphogenesis, the later stages were associated with cell expansion and the accumulation of storage reserves (mainly oil). There is strong evidence for the importance of FRKs in cell wall development. Thus, we infer that FRKs may be closely related to seed coat formation. In this study, the expression level of fructokinase (FRKS) (CmaCh17G006030) in CP variety was 7.49-fold times, which were higher than that in the HLCP variety on the 28th day after pollination, and its expression level was positively correlated with the lignin content in the seed coat, this results was consistent with the previous studies.

Sucrose synthase (SUS) was considered to be a central regulator of several metabolic and physiological processes during the process of plant growth and seed development (Abid et al., 2009). The importance of SUS in cell wall development, especially secondary cell wall development, has been demonstrated by overexpression of SUS in hybrid poplars (*Populus alba* × *grandidentata*). SUS enhanced cellulose synthesis and resulted in thicker secondary cell walls in xylem (Coleman et al., 2009). Ruan et al. (2008) found that the specific silent expression of SUS in cotton endosperm resulted in reduction of cell wall cellulose and corpus callosum significantly, which proved that SUS played an important role in the biosynthesis of cell wall in the endosperm of cotton seeds. Moreover, the overexpression of SUS gene of potato in cotton lead to fiber elongation and enhancement, which indicated that the importance of SUS in cell wall synthesis during cell division (Xu et al., 2012). These results indicated that the genes of starch and sucrose metabolism pathway may be closely related to the formation of seed coat. In this study, the expression level of SUS (CmaCh10G007940) in CP variety was 9.48- and 24.17-fold time, which was higher than that in HLCP variety on the 18th and 28th days after pollination.

### Transcription Factors Are Positively Involved in Seed Coat Formation

In recent years, many studies have shown that the regulation of gene transcription levels may be one of the most critical regulatory points in plant tissue development, and the different TFs that play a role in this process (Zhao and Dixon, 2011). bHLH TFs are one of the largest family of TFs in plants, and play an important role in signal transduction, growth and development, stress resistance, and other aspects of plants (Li et al., 2006). It has been found that during the heart-shaped development of *A. thaliana* embryos, the bHLH TF RGE1 in the endosperm play an important role in controlling embryo growth (Kondou et al., 2008). Antonio et al. (2016) found that the brown color of *A. thaliana* seeds is caused by the deposition of PAs (or condensed tannins) in the inner seed cortex; bHLH TFs complexes (such as TT2, TT8, and TTG1) regulate the expression of PA biosynthesis genes, and together regulate the development of trichomes in the bud epidermis and differentiation of mucus-producing cells in the exogenous cortex. Padmaja et al. (2014) found that TT8 gene mutation was necessary for inhibiting the transcription of mustard yellow seed mutant LBG and was responsible for regulating the color of the seed coat. Therefore, the bHLH TF family is indispensable for regulating the development of seed embryos, endosperms, and seed coats. In this study, there were significant differences in the expression levels of bHLH TFs between the two varieties. Three bHLH TFs were inferred to be related to the seed coat development of the CP variety, among which CYP92A6 was upregulated 224.29-times. However, the maximum upregulation of ELF2C was only 25.05-fold among the four bHLH TFs related to the HLCP variety of seed coat development. These results indicated that bHLH TFs are important TFs that may play an important role in the development of the seed coat. However, current research on bHLH TFs is still limited to model crops. Although bHLH-like TFs genes have been cloned from other plants, the functions of these genes need to be systematically studied. In addition to the bHLH TFs family, the MYB family is also involved in the regulation of seed coat morphogenesis.

Overexpression of MYB TFs genes (such as MYB46 and MYB83) in tobacco can stimulate the expression of genes related to cellulose, xylanase, and lignin biosynthesis, leading to abnormal thickening of secondary cell walls and deposition of lignin in some tissues (Zhong and Ye, 2012). Han (2014) used transcriptome high-throughput sequencing to screen the DEGs in soft-endocarp and hard-endocarp hawthorns, which indicated that *C*_4_*H*, *CCR*, and *F5H* are related to lignin biosynthesis, in addition to identifying that 12 MYB TFs and 4 NAC TFs are significantly downregulated in the soft-endocarp varieties. In this study, 25 MYB TFs candidate genes were identified from the DEGs in the CP variety. Among them, 14 genes were upregulated in the CP variety and 11 were downregulated in the HLCP variety. Fifteen MYB TF candidate genes were identified from the DEGs of the HLCP variety, of which nine genes were upregulated in hulled varieties and six genes were downregulated in the HLCP variety. This indicated that lignin in the seed coat is related to MYB TFs.

Zhong et al. (2008) showed that NAC TFs can activate MYB TFs in *A. thaliana* and jointly participate in the regulation of lignin accumulation and secondary wall formation in seeds. Zhu P. et al. (2016) found that NAC TFs in wheat can increase grain protein accumulation and promote the absorption of trace elements, such as zinc and iron. Uauy et al. (2006) found that NAC TFs (such as ttnam-b1, tnam-a1, and ttnam-b2) can promote the accumulation of protein and trace elements such as zinc and iron in grains to varying degrees. In this study, 13 NAC candidate genes were identified from the DEGs in the CP variety, of which six were upregulated in the CP variety and seven were downregulated in the HLCP variety. Four NAC candidate genes were identified from the DEGs in the HLCP variety. Four of these genes were upregulated in the CP variety and three were downregulated in the HLCP variety. Therefore, NAC TFs are important candidate genes for lignin biosynthesis in seed coats. In conclusion, MYB and NAC are important TFs in the lignin biosynthesis pathway, and thus, can be inferred to be closely related to the development of the HLCP seed coat.

### IRAK4 Proteins May Play a Positive Role in Seed Coat Development

IRAK4, a member of the IRAK family of intracellular serine threonine kinases, is involved in the process of immune response and apoptosis (Wang et al., 2006). At present, research on IRAK4 has mainly focused on medicine (Pacquelet et al., 2007; Guo et al., 2016). The IRAK family members have two special domains: an N-terminal conserved death domain and a kinase domain rich in serine and threonine (Janssens and Beyaert, 2003). Wang et al. (2017) found that IRAK4 has a positive effect on cardiomyocyte apoptosis. Sun et al. (2018) found that siRNA interfered with the expression of IRAK4 in cardiomyocytes induced by high glucose. The results showed that high glucose conditions significantly promoted the level of IRAK4 in cardiomyocytes, which could significantly reduce the viability of cardiomyocytes and increase the apoptosis rate. However, there are very few reports on the involvement of IRAK4 in plant seed growth and formation of seed coat. Xue et al. (2017) found that the genes related to lignin synthesis were highly expressed in hard-seed pomegranate. However, the expression of genes related to programmed cell death was slightly higher in soft-seed pomegranates. In this study, the results of the protein interaction network (PPI score >0.7) showed that IRAK4 interacted with 47 and 22 proteins during the development of CP and HLCP seed coats, respectively. Among them, IRAK4 (CmaCh14G016470) was upregulated 18.88-times in the CP variety, but not in the HLCP variety. IRAK4 (CmaCh04G023920) displayed the highest increase, 17.54-times in the CP variety, but only 1.58-times in the HLCP variety. This indicates that IRAK4 may be an important central protein, and its mechanism may be through the regulation of certain cell apoptosis during the development of the seed coat, so that the hypodermis and sclerenchyma tissues during the development of the seed coat of *C. pepo* are lost, leading to the formation of HLCP. Apoptosis is a type of programmed death caused by the activation of some coding genes in cells by inducing factors *in vivo* and *in vitro*. At present, it is generally believed that the expression of apoptosis-related genes is affected by some activated signaling pathways, which activate or synthesize a variety of enzymes that carry out apoptosis, thus leading to apoptosis. Therefore, based on the protein interaction network and the FPKM value of IRAK4, we speculate that IRAK4 protein may play a key role in the process of seed coat formation. In the future, we will conduct in-depth gene function research on how IRAK4 protein kinase plays an important role in seed coat development.

### Differentially Expressed Genes Are Involved in Signaling Pathways During the Development of the Seed Coat

**FIGURE 10:**
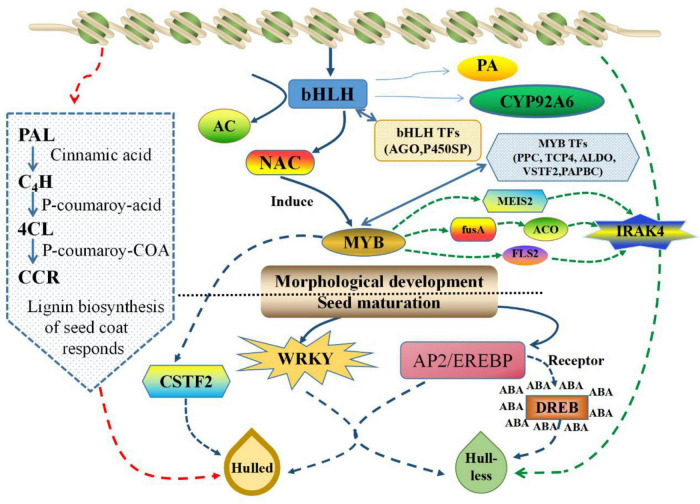
Summary of the molecular pathways and cellular processes involved in hull-less seed coat formation in *C. pepo*. The red line indicates the path of gene-level regulation, blue line represents the regulation of transcription factor level, and green line represents the action based on PPIs. The dashed arrows represent these genes that needs functional verification. The solid arrows indicate that the gene function has been reported.

The development of plant seeds is regulated by a series of related plant genes, TFs, enzymes, and proteins, which in combination with plant signal transduction pathways such as plant hormone signal transduction pathways, form a complex regulatory network that controls seed development (Gutierrez et al., 2007). Upon combining the results of histological observations and previous studies, we identified several major signal transduction pathways (Figure 10) that may be involved in the formation of the seed coat in *C. pepo*. First, the formation of the seed coat in HLCP occurs due to the lack of lignin accumulation. This factor is regulated by genes related to phenylalanine biosynthesis and metabolism pathways (such as *PAL*, *4CL*, and *CCR*). AC *cis*-acting elements are the most common promoters of phenylpropane biosynthesis genes. The coordinated expression of these genes is regulated by MYB TFs that bind to the AC elements. These changes may be caused by the co-regulation of TFs related to seed coat development (such as bHLH, NAC, MYB, WRKY, and AP2/EREBP). bHLH, NAC, and MYB jointly regulate the first stage of seed development and the morphological development process. The bHLH TF is mainly responsible for regulating the accumulation of PAs in the seed coat and regulating its color. MYB and NAC TFs jointly regulate lignin deposition and secondary wall formation, and MYB can be activated by NAC TFs. WRKY and AP2/EREBP TFs jointly regulate the second stage of seed development (i.e., seed maturation).

In addition, MYB TFs can also regulate downstream IRAK4 protein kinases through MEIS2, fusA, ACO, and FLS2, which may lead to a lack of hypodermis and sclerenchyma during seed development by regulating cell apoptosis during seed coat development. Finally, the formation of seed coat in HLCP may be controlled by a main effect gene and multiple miniature effect genes. The function of many DEGs is still unknown, and some candidate genes have been identified in the NCBI database. For example, pure acid phosphate (CSTF) may play an important role in the MYB pathway, IAA may play an important role in the bHLH pathway, and IRAK4 plays an important role in the formation of the naked seed coat in the signal transduction process. In addition, most DEGs related to seed coat formation were enriched in the phenylpropanoid biosynthesis pathway. In summary, these results provide great inspiration for future research on the seed coat of *C. pepo* L.

## Data Availability Statement

The datasets presented in this study can be found in online repositories. The names of the repository/repositories and accession number(s) can be found in the article/Supplementary Material.

## Author Contributions

BX and YX developed this concept and received financial support. YX, FT, and ZS designed the experiments. ZS performed the experiments, analyzed the data, and wrote the manuscript. FT and JZ modified the manuscript. All authors contributed to the article and approved the submitted version.

## Conflict of Interest

The authors declare that the research was conducted in the absence of any commercial or financial relationships that could be construed as a potential conflict of interest.

## Publisher’s Note

All claims expressed in this article are solely those of the authors and do not necessarily represent those of their affiliated organizations, or those of the publisher, the editors and the reviewers. Any product that may be evaluated in this article, or claim that may be made by its manufacturer, is not guaranteed or endorsed by the publisher.

## Footnotes

1
http://www.ccb.jhu.edu/software/hisat

2
http://cucurbitgenomics.org/organism/8

3
http://ccb.jhu.edu/software/stringtie

4
http://cole-trapnell-lab.github.io/cufflinks

5
http://cpc.cbi.pku.edu.cn

6
http://emboss.sourceforge.net/apps/cvs/emboss/apps/getorf.html

7
http://hmmer.org

8
http://plntfdb.bio.uni-potsdam.de/v3.0/

9
http://string-db.org/
