# Supplementary material for: Transcriptomic Analysis Reveal the Molecular Mechanisms of Seed Coat Development in Cucurbita pepo L
Source: Front Plant Sci. 2022 Feb 24;13:772685. doi: 10.3389/fpls.2022.772685 (PMC8912962; doi:10.3389/fpls.2022.772685)
Supplement: Supplementary file 2 [file Data_Sheet_2.DOCX]

| **Supplementary Table 1.** Primers used for RT-qPCR. | | | | | | | | |
| --- | --- | --- | --- | --- | --- | --- | --- | --- |
| **Gene Name** | | **Sequence** | **Length** | **Gene Length (bp)** | **Position (mRNA)** | **Tm**（**℃**） | **GC %** | **Product Length (bp)** |
| UDP-glycosyltransferase  72E1-like | -F | 5’-CCTCCACCACAACCTCCAA -3’ | 19 | 1451 | 213 | 61.95 | 57.89 | 120 |
|  | -R | 5’-GAGGAGTGACGAATAGGGACGA -3’ | 22 |  | 332 | 62.77 | 54.55 |  |
| Alpha/beta-Hydrolases  superfamily protein | -F | 5’-CATCTCCCTGCCTTCCTCTTC -3’ | 21 | 975 | 391 | 62.51 | 57.14 | 144 |
|  | -R | 5’-TTTGCTCGGCTTCATGTTTTC -3’ | 21 |  | 534 | 62.49 | 42.86 |  |
| Laccase | -F | 5’-ATATTCTATGGCTGAGGGCGACT -3’ | 23 | 1942 | 377 | 62.41 | 47.83 | 90 |
|  | -R | 5’-CTTTGTGAGGAGCAGGGAATG -3’ | 21 |  | 466 | 62.07 | 52.38 |  |
| Aspartic proteinase | -F | 5’-CATTGGCACTCCACCTCAAA -3’ | 20 | 1896 | 405 | 62.04 | 50.00 | 98 |
|  | -R | 5’-AGCAAGCAATCGAGAAAACACA -3’ | 22 |  | 502 | 62.18 | 40.91 |  |
| Calcium-transporting ATPase 2 | -F | 5’-AGTGAAGGTGGAGATGACGAAAC -3’ | 23 | 3867 | 1513 | 61.79 | 47.83 | 94 |
|  | -R | 5’-TGATGATGGCAAAGAACAGACC -3’ | 22 |  | 1606 | 62.33 | 45.45 |  |
| Galactosylgalactosylxylosylprotein 3-beta-glucuronosyltransferase 1 | -F | 5’-AATGTGAGGTGGTTTGGTGCT -3’ | 21 | 1518 | 832 | 61.74 | 47.62 | 98 |
|  | -R | 5’-GCAGGAATTGGAAGGTTCTCC -3’ | 21 |  | 929 | 62.19 | 52.38 |  |
| Mitochondrial carrier | -F | 5’-GGTGGAGCTGGTGAAAATCC -3’ | 20 | 1068 | 375 | 61.80 | 55.00 | 115 |
|  | -R | 5’-TCCTCTGTAAATCCCTCTCAATCC -3’ | 24 |  | 489 | 61.94 | 45.83 |  |
| Cellulose synthase | -F | 5’-CTCTGCTAATCCCTCCCACAAC -3’ | 22 | 3546 | 2915 | 62.23 | 54.55 | 136 |
|  | -R | 5’-ATCACCCAGAAGGCAAAGAAAA -3’ | 22 |  | 3050 | 62.14 | 40.91 |  |
| Caffeoyl-CoA O-  methyltransferase | -F | 5’-TCCACTACCGTCGCACAAG -3’ | 19 | 1008 | 69 | 60.88 | 57.89 | 133 |
|  | -R | 5’-GTTCGGGCTCTCTTGGATAAA -3’ | 21 |  | 201 | 60.57 | 47.62 |  |
| Protein NRT1/ PTR  FAMILY 3.1 | -F | 5’-ACTCCAACAGGGCACAACC -3’ | 19 | 2118 | 1191 | 60.98 | 57.89 | 136 |
|  | -R | 5’-GGCGATGGGGATGAAGA -3’ | 17 |  | 1326 | 60.54 | 58.82 |  |
| Adenine  phosphoribosyltransferase | -F | 5’-AACCATCATCCCTTTCAACCTCT -3’ | 23 | 1296 | 785 | 62.24 | 43.48 | 121 |
|  | -R | 5’-TACGGCTCCTACCCAAACTCAC -3’ | 22 |  | 905 | 62.59 | 54.55 |  |
| Secondary cell wall-related glycosyltransferase family 47 | -F | 5’-GAAAGAGATGAGGGTTTTCGTGT -3’ | 23 | 1757 | 261 | 60.86 | 43.48 | 116 |
|  | -R | 5’-GGGCTCTATGAATGGCTACCTC -3’ | 22 |  | 376 | 61.29 | 54.55 |  |
| **XM_023673479.1 (**β-Act**in)** | -F | 5’- CAAGAACTGGAGACAGCCAAGA -3’ | 22 | 1927 | 845 | 61.86 | 55.56 | 249 |
|  | -R | 5’- GAACATCGTTGACCCACCAC -3’ | 20 |  | 1090 | 61.25 | 44.00 |  |
| **MH310440.1 (**α-tub**ulin)** | -F | 5’-GCGTTTATCGGTTGACTATGGAA -3’ | 23 | 1863 | 689 | 62.33 | 43.48 | 116 |
|  | -R | 5’-GGGAATGGGTTGAGAGGACA -3’ | 20 |  | 804 | 62.23 | 55.00 |  |

**Supplementary Table 2.** Results of total RNA sample quality tests of 18 samples.

| **Sample** | **Concentration (ng/ μL)** | **Volume (μL)** | **OD260/ 280** | **OD260/ 230** | **RIN** | **28S/ 18S** | **Platform Type** |
| --- | --- | --- | --- | --- | --- | --- | --- |
| CP8-1 | 1000 | 48 | 2.1 | 2.3 | 8.2 | 1.5 | BGISEQ-500 Transcriptome |
| CP8-2 | 872 | 49 | 2.09 | 2.21 | 8.4 | 1.6 | BGISEQ-500 Transcriptome |
| CP8-3 | 965 | 50 | 2.11 | 2.27 | 8.1 | 1.5 | BGISEQ-500 Transcriptome |
| CP18-1 | 624 | 49 | 2.12 | 2.26 | 8.1 | 1.5 | BGISEQ-500 Transcriptome |
| CP18-2 | 1100 | 49 | 2.12 | 2.31 | 8.4 | 1.5 | BGISEQ-500 Transcriptome |
| CP18-3 | 576 | 49 | 2.06 | 2.24 | 7.9 | 1.6 | BGISEQ-500 Transcriptome |
| CP28-1 | 288 | 46 | 2.14 | 2.28 | 7.8 | 1.7 | BGISEQ-500 Transcriptome |
| CP28-2 | 127 | 47 | 2.08 | 2.14 | 7.3 | 1.4 | BGISEQ-500 Transcriptome |
| CP28-3 | 296 | 47 | 2.1 | 2.23 | 7.7 | 1.5 | BGISEQ-500 Transcriptome |
| HLCP8-1 | 1928 | 51 | 2.1 | 2.31 | 9.1 | 1.7 | BGISEQ-500 Transcriptome |
| HLCP8-2 | 2331 | 48 | 2.07 | 2.29 | 9.0 | 1.6 | BGISEQ-500 Transcriptome |
| HLCP8-3 | 1544 | 47 | 2.1 | 2.33 | 8.9 | 1.6 | BGISEQ-500 Transcriptome |
| HLCP18-1 | 675 | 49 | 2.12 | 2.23 | 7.6 | 1.3 | BGISEQ-500 Transcriptome |
| HLCP18-2 | 723 | 49 | 1.92 | 2.14 | 7.4 | 1.4 | BGISEQ-500 Transcriptome |
| HLCP18-3 | 1155 | 49 | 2.11 | 2.3 | 8.2 | 1.5 | BGISEQ-500 Transcriptome |
| HLCP28-1 | 843 | 47 | 2.11 | 2.27 | 7.7 | 1.5 | BGISEQ-500 Transcriptome |
| HLCP28-2 | 1252 | 47 | 2.12 | 2.29 | 8.0 | 1.5 | BGISEQ-500 Transcriptome |
| HLCP28-3 | 840 | 47 | 2.1 | 2.25 | 7.8 | 1.6 | BGISEQ-500 Transcriptome |

**Supplementary Table 3.** Quality statistics of filtered reads.

| **Sample name** | **Total Raw Reads (M)** | **Total Clean Reads (M)** | **Total RNA RIN** | **Clean Reads Q30(%)** | **Clean Reads Ratio(%)** |
| --- | --- | --- | --- | --- | --- |
| CP181 | 115.39 | 110.33 | 8.2 | 91.27 | 95.62 |
| CP182 | 115.39 | 109.75 | 8.4 | 91.29 | 95.11 |
| CP183 | 112.88 | 107.58 | 8.1 | 91.49 | 95.3 |
| CP281 | 112.89 | 108.04 | 8.1 | 88.07 | 95.7 |
| CP282 | 112.89 | 108.07 | 8.4 | 88.15 | 95.73 |
| CP283 | 112.89 | 108.11 | 7.9 | 87.97 | 95.76 |
| CP81 | 115.39 | 110.15 | 7.8 | 91.34 | 95.46 |
| CP82 | 117.9 | 111.86 | 7.3 | 91.48 | 94.88 |
| CP83 | 112.88 | 107.74 | 7.7 | 91.3 | 95.45 |
| HLCP181 | 115.4 | 110.46 | 9.1 | 88.13 | 95.72 |
| HLCP182 | 115.4 | 110.65 | 9.0 | 87.94 | 95.89 |
| HLCP183 | 110.38 | 105.69 | 8.9 | 88.18 | 95.75 |
| HLCP281 | 112.89 | 108 | 7.6 | 88.18 | 95.67 |
| HLCP282 | 112.89 | 107.7 | 7.4 | 88.2 | 95.4 |
| HLCP283 | 112.89 | 107.38 | 8.2 | 88.24 | 95.12 |
| HLCP81 | 112.89 | 108.3 | 7.7 | 88.14 | 95.93 |
| HLCP82 | 115.4 | 110.01 | 8.0 | 88.08 | 95.33 |
| HLCP83 | 112.89 | 107.77 | 7.8 | 87.96 | 95.46 |

**Supplementary Table 4.** Square of Pearson correlation coefficient (R^2^).

| **Samples** | **Person** | **R^2^** |
| --- | --- | --- |
| CP81 VS CP82 | 0.9750205 | 0.9507 |
| CP82 VS CP83 | 0.9788159 | 0.9581 |
| CP81 VS CP83 | 0.9543449 | 0.9108 |
| CP181 VS CP182 | 0.9884212 | 0.977 |
| CP182 VS CP183 | 0.9752906 | 0.9512 |
| CP181 VS CP183 | 0.9902903 | 0.9807 |
| CP281 VS CP282 | 0.9575054 | 0.9168 |
| CP282 VS CP283 | 0.9836303 | 0.9675 |
| CP281 VS CP283 | 0.9578099 | 0.9174 |
| HLCP81 VS HLCP82 | 0.9711793 | 0.9432 |
| HLCP82 VS HLCP83 | 0.9525841 | 0.9074 |
| HLCP81 VS HLCP83 | 0.9774756 | 0.9555 |
| HLCP181 VS HLCP182 | 0.9927994 | 0.9857 |
| HLCP182 VS HLCP183 | 0.981843 | 0.964 |
| HLCP181 VS HLCP183 | 0.9773405 | 0.9552 |
| HLCP281 VS HLCP282 | 0.9872857 | 0.9747 |
| HLCP282 VS HLCP283 | 0.9714038 | 0.9436 |
| HLCP281 VS HLCP283 | 0.9615618 | 0.9246 |
| **AVE** | **0.974144567** | **0.9491** |

**Supplementary Table 5.** Statistics of reference genome alignment results.

| **Sample** | **Total Clean Reads (M)** | **Total Mapping (%)** | **Uniquely Mapping (%)** |
| --- | --- | --- | --- |
| CP181 | 110.33 | 51.61 | 31.93 |
| CP182 | 109.75 | 53.41 | 33.04 |
| CP183 | 107.58 | 50.83 | 31.48 |
| CP281 | 108.04 | 49.37 | 29.89 |
| CP282 | 108.07 | 50.68 | 30.61 |
| CP283 | 108.11 | 50.73 | 30.6 |
| CP81 | 110.15 | 67.32 | 42.15 |
| CP82 | 111.86 | 67.51 | 42.36 |
| CP83 | 107.74 | 67.54 | 42.33 |
| HLCP181 | 110.46 | 44.2 | 26.74 |
| HLCP182 | 110.65 | 44.61 | 26.9 |
| HLCP183 | 105.69 | 47.32 | 28.5 |
| HLCP281 | 108 | 49.62 | 29.64 |
| HLCP282 | 107.7 | 51.03 | 30.52 |
| HLCP283 | 107.38 | 51.7 | 31.03 |
| HLCP81 | 108.3 | 64.79 | 39.64 |
| HLCP82 | 110.01 | 64.89 | 39.59 |
| HLCP83 | 107.77 | 64.65 | 39.35 |
| Average |  | 55.1 |  |

**Supplementary Table 6.** Statistics of reference gene alignment results

| **Sample** | **Total Clean Reads (M)** | **Total Mapping (%)** | **Uniquely Mapping (%)** |
| --- | --- | --- | --- |
| CP181 | 110.33 | 54.03 | 24.13 |
| CP182 | 109.75 | 55.88 | 25.1 |
| CP183 | 107.58 | 53.26 | 23.84 |
| CP281 | 108.04 | 52.76 | 23.98 |
| CP282 | 108.07 | 54.3 | 24.3 |
| CP283 | 108.11 | 54.51 | 24.35 |
| CP81 | 110.15 | 67.95 | 31.52 |
| CP82 | 111.86 | 68.4 | 31.57 |
| CP83 | 107.74 | 68.4 | 31.75 |
| HLCP181 | 110.46 | 47.02 | 21.51 |
| HLCP182 | 110.65 | 47.72 | 22.03 |
| HLCP183 | 105.69 | 50.65 | 23.12 |
| HLCP281 | 108 | 53.22 | 23.66 |
| HLCP282 | 107.7 | 54.81 | 24.34 |
| HLCP283 | 107.38 | 55.59 | 24.86 |
| HLCP81 | 108.3 | 67.27 | 31.82 |
| HLCP82 | 110.01 | 67.3 | 31.81 |
| HLCP83 | 107.77 | 67.58 | 32.08 |
| Average |  | 57.81 |  |

**Supplementary Table 7.** Statistics of new transcripts.

| **Total Novel Transcript** | **Coding Transcript** | **Noncoding Transcript** | **Novel Isoform** | **Novel Gene** |
| --- | --- | --- | --- | --- |
| 18332 | 17314 | 1018 | 16958 | 356 |

| **Supplementary Table 8.** GO enrichment of 2099 DEGs in hulled *C. pepo*. | | | | | | | | |
| --- | --- | --- | --- | --- | --- | --- | --- | --- |
|  | **GO Term ID** | **GO Term** | **Term Candidate Gene Num** | **Total Candidate Gene Num** | **Term Gene Num** | **Total Gene Num** | **Rich Ratio** | **Q value** |
| **Biological process** | GO:0009698 | phenylpropanoid metabolic process | 12 | 711 | 51 | 12668 | 23.53% | 0.007724889 |
|  | GO:0042537 | benzene-containing compound metabolic process | 7 | 711 | 20 | 12668 | 35.00% | 0.01721375 |
|  | GO:0000271 | polysaccharide biosynthetic process | 24 | 711 | 186 | 12668 | 12.90% | 0.020540005 |
|  | GO:0009063 | cellular amino acid catabolic process | 11 | 711 | 53 | 12668 | 20.75% | 0.020540005 |
|  | GO:0009074 | aromatic amino acid family catabolic process | 7 | 711 | 21 | 12668 | 33.33% | 0.020540005 |
|  | GO:0033692 | cellular polysaccharide biosynthetic process | 20 | 711 | 143 | 12668 | 13.99% | 0.020540005 |
|  | GO:0044264 | cellular polysaccharide metabolic process | 28 | 711 | 235 | 12668 | 11.91% | 0.020540005 |
|  | GO:0009072 | aromatic amino acid family metabolic process | 13 | 711 | 73 | 12668 | 17.81% | 0.022772715 |
|  | GO:1901606 | alpha-amino acid catabolic process | 10 | 711 | 48 | 12668 | 20.83% | 0.027029345 |
|  | GO:0009699 | phenylpropanoid biosynthetic process | 6 | 711 | 18 | 12668 | 33.33% | 0.027545401 |
|  | GO:0009800 | cinnamic acid biosynthetic process | 6 | 711 | 18 | 12668 | 33.33% | 0.027545401 |
|  | GO:0009803 | cinnamic acid metabolic process | 6 | 711 | 18 | 12668 | 33.33% | 0.027545401 |
|  | GO:0006559 | L-phenylalanine catabolic process | 6 | 711 | 19 | 12668 | 31.58% | 0.031747147 |
|  | GO:1902222 | erythrose 4-phosphate/phosphoenolpyruvate family amino acid catabolic process | 6 | 711 | 19 | 12668 | 31.58% | 0.031747147 |
|  | GO:1901605 | alpha-amino acid metabolic process | 29 | 711 | 267 | 12668 | 10.86% | 0.034348892 |
|  | GO:0030244 | cellulose biosynthetic process | 12 | 711 | 71 | 12668 | 16.90% | 0.034949466 |
|  | GO:0042737 | drug catabolic process | 29 | 711 | 268 | 12668 | 10.82% | 0.034949466 |
|  | GO:0019748 | secondary metabolic process | 12 | 711 | 72 | 12668 | 16.67% | 0.038634562 |
|  | GO:0006558 | L-phenylalanine metabolic process | 7 | 711 | 29 | 12668 | 24.14% | 0.047915206 |
|  | GO:0044262 | cellular carbohydrate metabolic process | 32 | 711 | 316 | 12668 | 10.13% | 0.047915206 |
|  | GO:1902221 | erythrose 4-phosphate/phosphoenolpyruvate family amino acid metabolic process | 7 | 711 | 29 | 12668 | 24.14% | 0.047915206 |
| **Cellular component** | GO:0016021 | integral component of membrane | 564 | 918 | 8468 | 15910 | 6.66% | 0.000150148 |
|  | GO:0031224 | intrinsic component of membrane | 566 | 918 | 8483 | 15910 | 6.67% | 0.000150148 |
|  | GO:0044425 | membrane part | 570 | 918 | 8769 | 15910 | 6.50% | 0.003261635 |
|  | GO:0016020 | membrane | 582 | 918 | 9128 | 15910 | 6.38% | 0.01721375 |
| **Molecular function** | GO:0020037 | heme binding | 51 | 1227 | 458 | 21381 | 11.14% | 0.003229508 |
|  | GO:0016841 | ammonia-lyase activity | 8 | 1227 | 23 | 21381 | 34.78% | 0.007724889 |
|  | GO:0046906 | tetrapyrrole binding | 55 | 1227 | 538 | 21381 | 10.22% | 0.007724889 |
|  | GO:0016705 | oxidoreductase activity, acting on paired donors, with incorporation or reduction of molecular oxygen | 41 | 1227 | 391 | 21381 | 10.49% | 0.020540005 |
|  | GO:0004672 | protein kinase activity | 139 | 1227 | 1797 | 21381 | 7.74% | 0.020609697 |
|  | GO:0016759 | cellulose synthase activity | 11 | 1227 | 54 | 21381 | 20.37% | 0.022772715 |
|  | GO:0016760 | cellulose synthase (UDP-forming) activity | 11 | 1227 | 54 | 21381 | 20.37% | 0.022772715 |
|  | GO:0004497 | monooxygenase activity | 36 | 1227 | 337 | 21381 | 10.68% | 0.02660542 |
|  | GO:0022857 | transmembrane transporter activity | 83 | 1227 | 992 | 21381 | 8.37% | 0.028854998 |
|  | GO:0045548 | phenylalanine ammonia-lyase activity | 6 | 1227 | 18 | 21381 | 33.33% | 0.028854998 |
|  | GO:0016840 | carbon-nitrogen lyase activity | 9 | 1227 | 41 | 21381 | 21.95% | 0.031747147 |
|  | GO:0016757 | transferase activity, transferring glycosyl groups | 66 | 1227 | 766 | 21381 | 8.62% | 0.039542165 |
|  | GO:0016773 | phosphotransferase activity, alcohol group as acceptor | 149 | 1227 | 2020 | 21381 | 7.38% | 0.045184067 |
|  | GO:0005506 | iron ion binding | 41 | 1227 | 426 | 21381 | 9.62% | 0.047915206 |

| **Supplementary Table 9.** GO enrichment of 1831 DEGs in hull-less *C. pepo* L. | | | | | | | | |
| --- | --- | --- | --- | --- | --- | --- | --- | --- |
|  | **GO Term ID** | **GO Term** | **Term Candidate Gene Num** | **Total Candidate Gene Num** | **Term Gene Num** | **Total Gene Num** | **Rich Ratio** | **Q value** |
| Biological process | GO:0005975 | carbohydrate metabolic process | 95 | 628 | 1267 | 12668 | 0.074980268 | 0.0055158 |
|  | GO:0007017 | microtubule-based process | 26 | 628 | 214 | 12668 | 0.121495327 | 0.0055158 |
|  | GO:0006828 | manganese ion transport | 3 | 628 | 3 | 12668 | 1 | 0.01253124 |
|  | GO:0006880 | intracellular sequestering of iron ion | 3 | 628 | 3 | 12668 | 1 | 0.01253124 |
|  | GO:0030026 | cellular manganese ion homeostasis | 3 | 628 | 3 | 12668 | 1 | 0.01253124 |
|  | GO:0051238 | sequestering of metal ion | 3 | 628 | 3 | 12668 | 1 | 0.01253124 |
|  | GO:0055071 | manganese ion homeostasis | 3 | 628 | 3 | 12668 | 1 | 0.01253124 |
|  | GO:0071421 | manganese ion transmembrane transport | 3 | 628 | 3 | 12668 | 1 | 0.01253124 |
|  | GO:0097577 | sequestering of iron ion | 3 | 628 | 3 | 12668 | 1 | 0.01253124 |
|  | GO:0006928 | movement of cell or subcellular component | 15 | 628 | 118 | 12668 | 0.127118644 | 0.044853272 |
|  | GO:0007018 | microtubule-based movement | 15 | 628 | 118 | 12668 | 0.127118644 | 0.044853272 |
| Cellular component | GO:0016021 | integral component of membrane | 487 | 791 | 8468 | 15910 | 0.057510628 | 0.000972755 |
|  | GO:0031224 | intrinsic component of membrane | 487 | 791 | 8483 | 15910 | 0.057408936 | 0.000972755 |
|  | GO:0044425 | membrane part | 495 | 791 | 8769 | 15910 | 0.056448854 | 0.003919028 |
|  | GO:0016020 | membrane | 510 | 791 | 9128 | 15910 | 0.055872042 | 0.0055158 |
| Molecular function | GO:0003824 | catalytic activity | 710 | 1070 | 12813 | 21381 | 0.055412472 | 0.003428821 |
|  | GO:0005506 | iron ion binding | 43 | 1070 | 426 | 21381 | 0.100938967 | 0.004205472 |
|  | GO:0020037 | heme binding | 44 | 1070 | 458 | 21381 | 0.096069869 | 0.006280279 |
|  | GO:0016798 | hydrolase activity, acting on glycosyl bonds | 57 | 1070 | 666 | 21381 | 0.085585586 | 0.011467778 |
|  | GO:0004030 | aldehyde dehydrogenase [NAD(P)+] activity | 5 | 1070 | 11 | 21381 | 0.454545455 | 0.01253124 |
|  | GO:0005384 | manganese ion transmembrane transporter activity | 3 | 1070 | 3 | 21381 | 1 | 0.01253124 |
|  | GO:0016705 | oxidoreductase activity, acting on paired donors, with incorporation or reduction of molecular oxygen | 38 | 1070 | 391 | 21381 | 0.097186701 | 0.01253124 |
|  | GO:0046906 | tetrapyrrole binding | 47 | 1070 | 538 | 21381 | 0.087360595 | 0.014885196 |
|  | GO:0004553 | hydrolase activity, hydrolyzing O-glycosyl compounds | 53 | 1070 | 634 | 21381 | 0.083596215 | 0.016960368 |
|  | GO:0016740 | transferase activity | 303 | 1070 | 5101 | 21381 | 0.059400118 | 0.027589375 |
|  | GO:0008017 | microtubule binding | 21 | 1070 | 183 | 21381 | 0.114754098 | 0.02869282 |
|  | GO:0015631 | tubulin binding | 22 | 1070 | 197 | 21381 | 0.111675127 | 0.029534203 |
|  | GO:0004497 | monooxygenase activity | 32 | 1070 | 337 | 21381 | 0.09495549 | 0.03040636 |
|  | GO:0016773 | phosphotransferase activity, alcohol group as acceptor | 134 | 1070 | 2020 | 21381 | 0.066336634 | 0.03040636 |
|  | GO:0003854 | 3-beta-hydroxy-delta5-steroid dehydrogenase activity | 4 | 1070 | 9 | 21381 | 0.444444444 | 0.043206901 |
|  | GO:0004672 | protein kinase activity | 120 | 1070 | 1797 | 21381 | 0.066777963 | 0.043206901 |
|  | GO:0033764 | steroid dehydrogenase activity, acting on the CH-OH group of donors, NAD or NADP as acceptor | 4 | 1070 | 9 | 21381 | 0.444444444 | 0.043206901 |
|  | GO:0003774 | motor activity | 17 | 1070 | 142 | 21381 | 0.11971831 | 0.045762885 |
|  | GO:0003777 | microtubule motor activity | 15 | 1070 | 118 | 21381 | 0.127118644 | 0.047772245 |
