# Supplementary material for: Transcriptomic Analysis Reveal the Molecular Mechanisms of Seed Coat Development in Cucurbita pepo L
Source: Front Plant Sci. 2022 Feb 24;13:772685. doi: 10.3389/fpls.2022.772685 (PMC8912962; doi:10.3389/fpls.2022.772685)
Supplement: Supplementary file 6 [file Data_Sheet_6.DOCX]

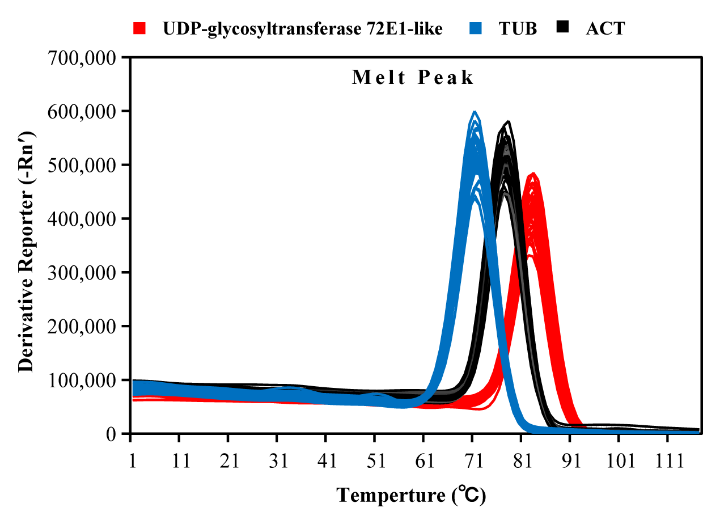

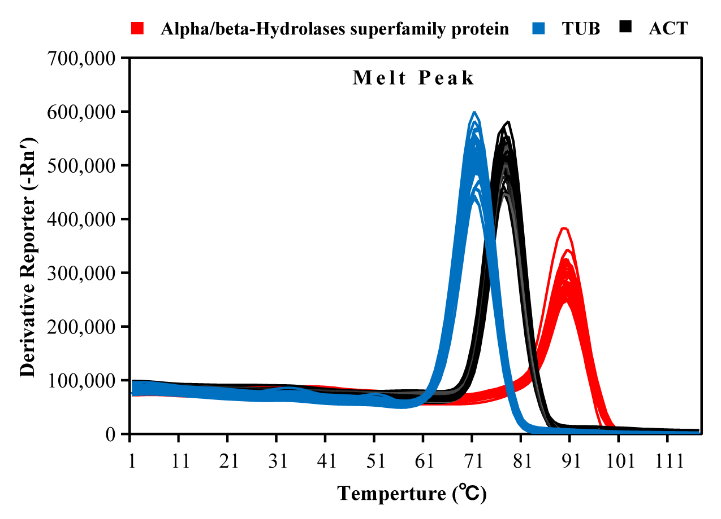

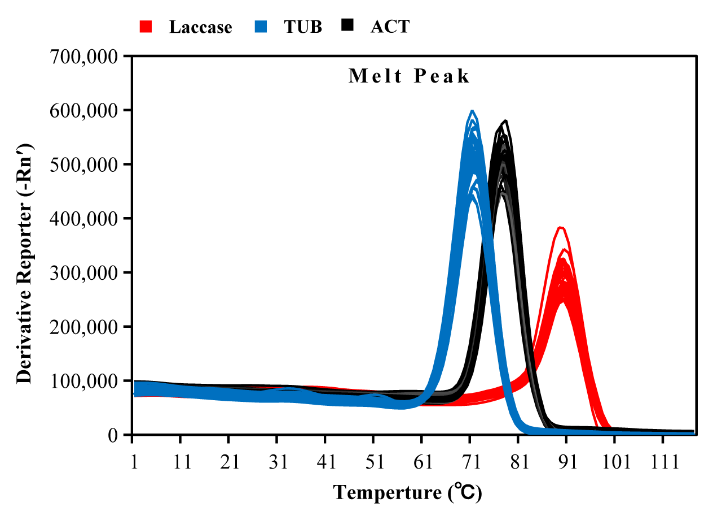

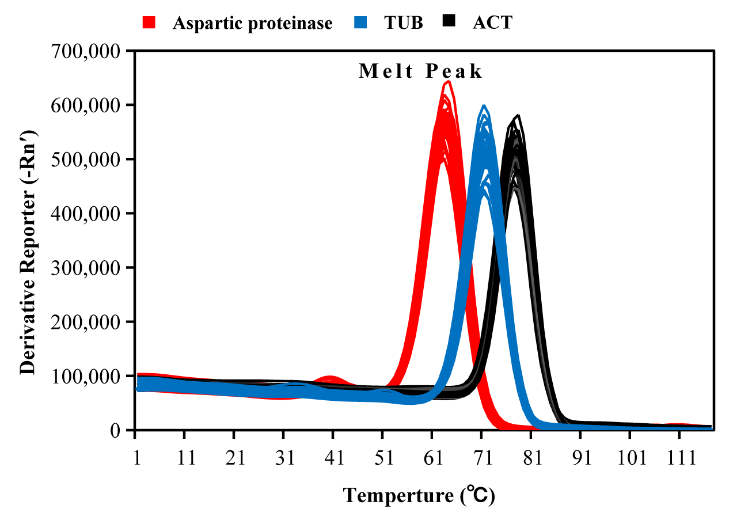

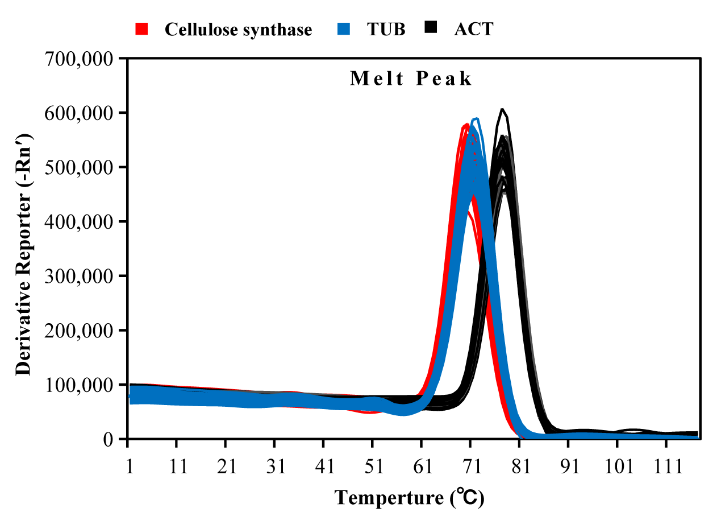

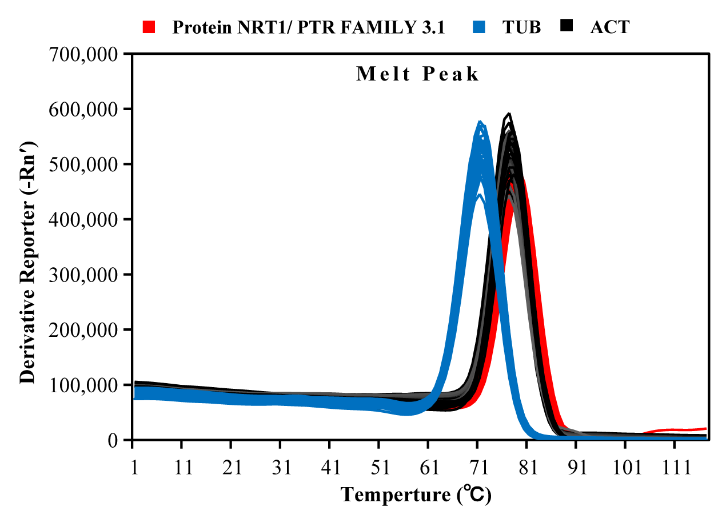

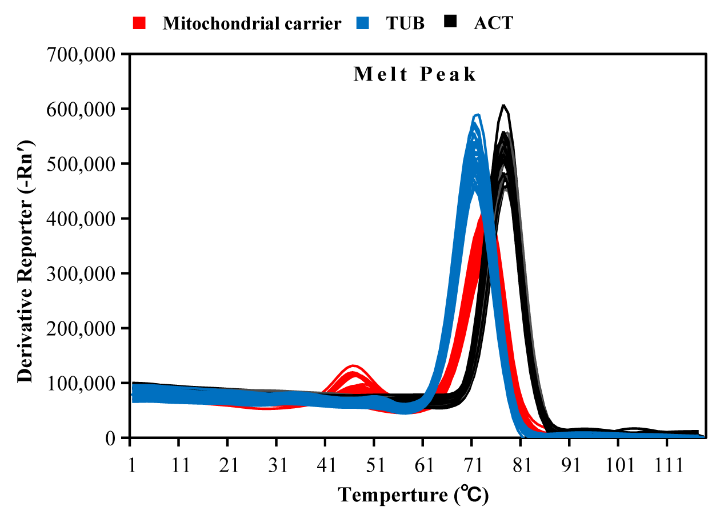

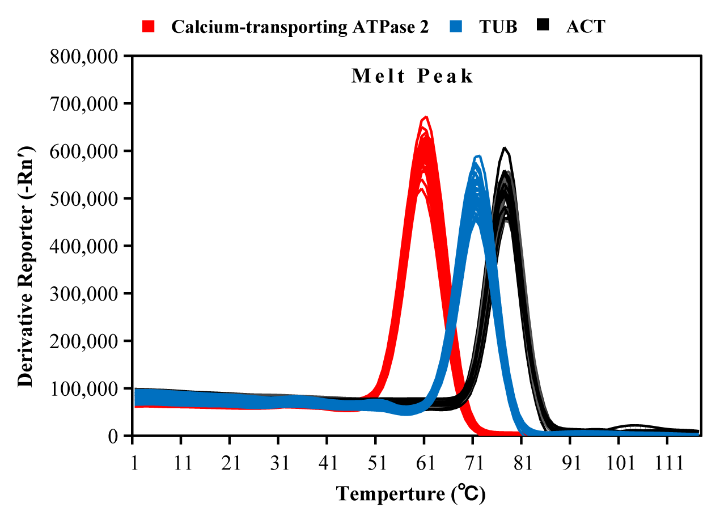

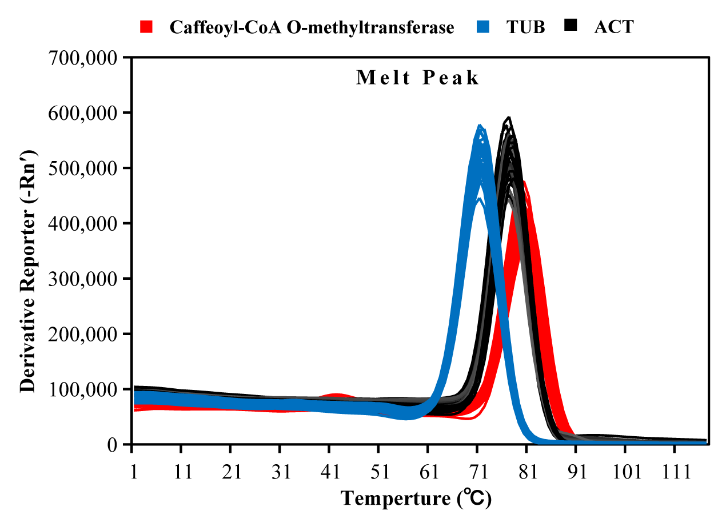

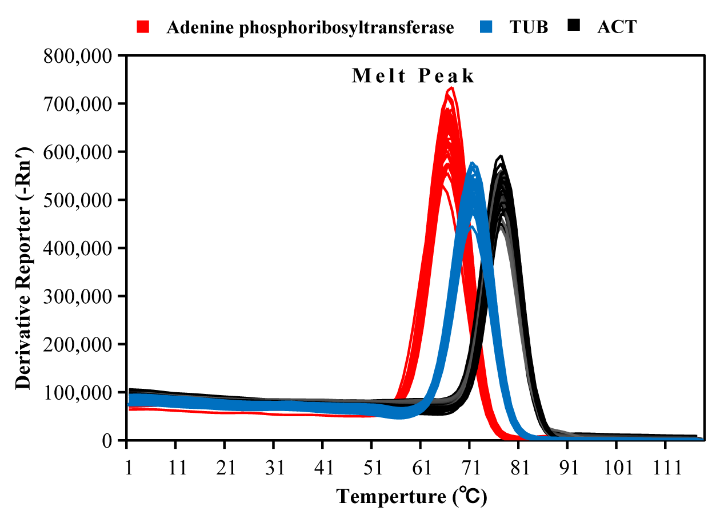

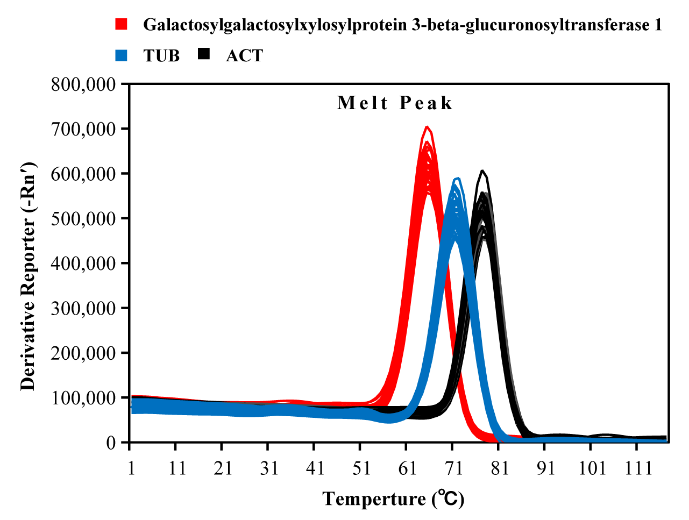

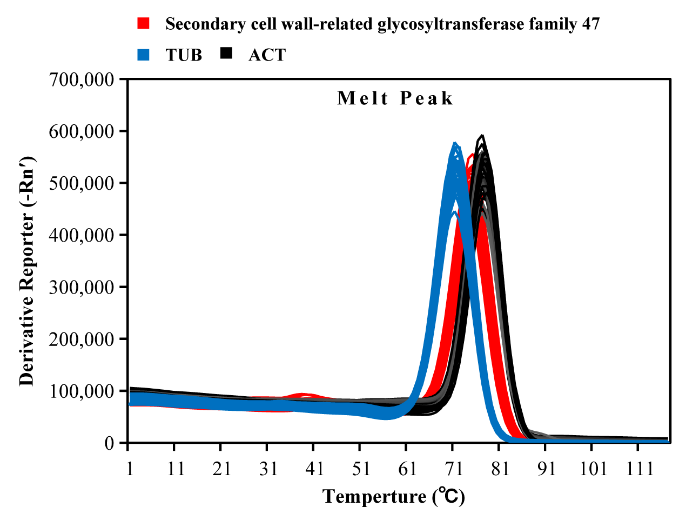


**Figure S1.** Melting curves of each transcript. *α-tubulin* (TUB, blue) and *β-actin* (ACT, black) genes, which were chosen as reference genes. Red represents the target gene.

**
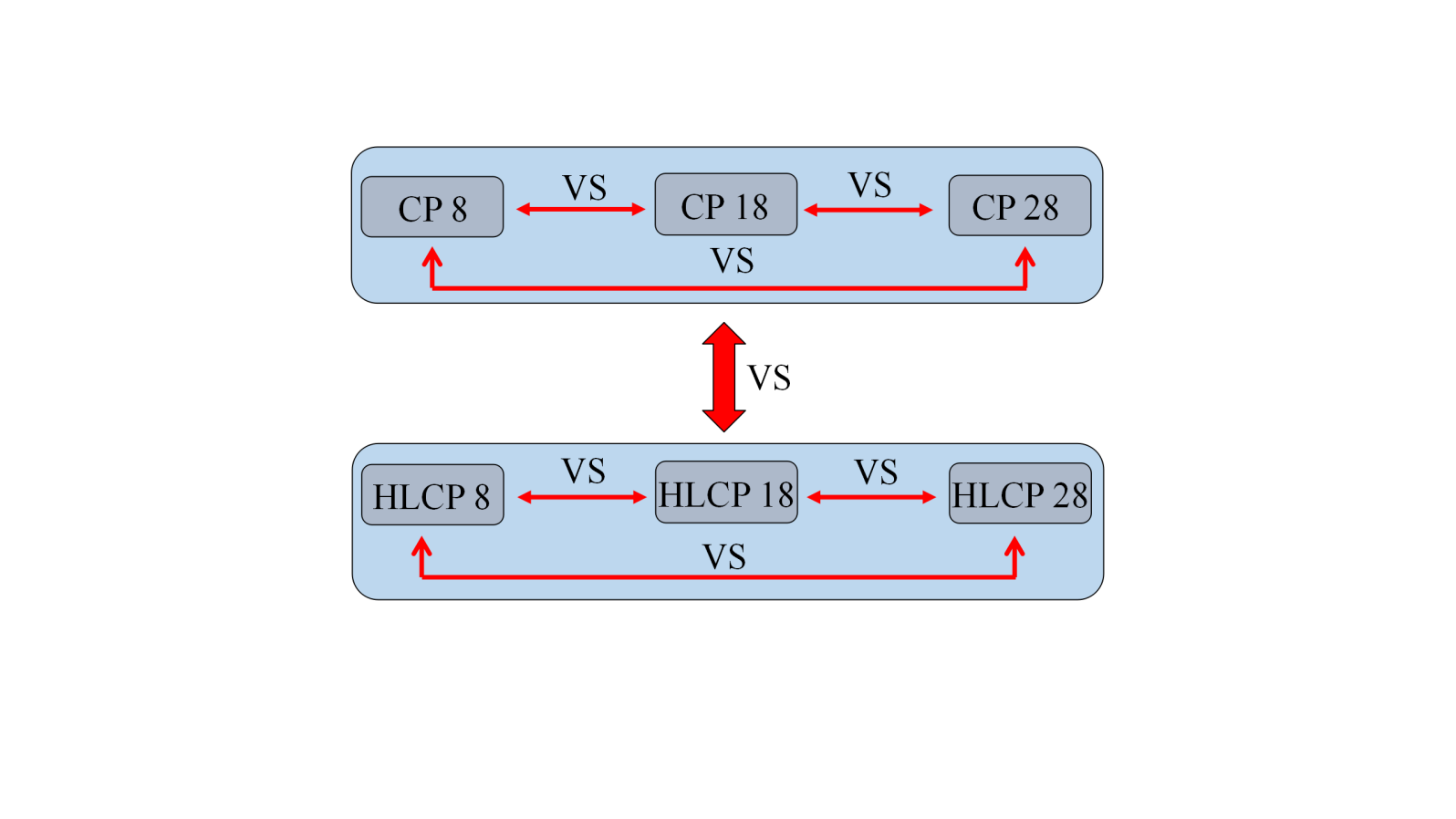
**

**Figure S2.** Analysis for identification of DEGs in the seed coat of hulled and hull-less *C. pepo*. CP represents hulled *C. pepo.* HLCP represents hull-less *C. pepo* L. First, differences in gene expression in three stages (8, 18, and 28 d post-pollination) of CP and HLCP were analyzed: CP8 *vs.* CP18, CP18 *vs.* CP28, and CP8 *vs.* CP28, as well as, HLCP8 *vs.* HLCP18, HLCP18 *vs.* HLCP28, and HLCP8 *vs.* HLCP28. Then, the same was carried out again to identify unique DEGs between CP and HLCP. The results of the comparison identified that the DEGs of the hulled variety are genes related to the development of the seed coat of CP, while the DEGs of the hull-less variety are genes related to the development of the seed coat of HLCP. CK: The seed coats of HLCP on the 8th day after pollination were used as controls for CP and HLCP.


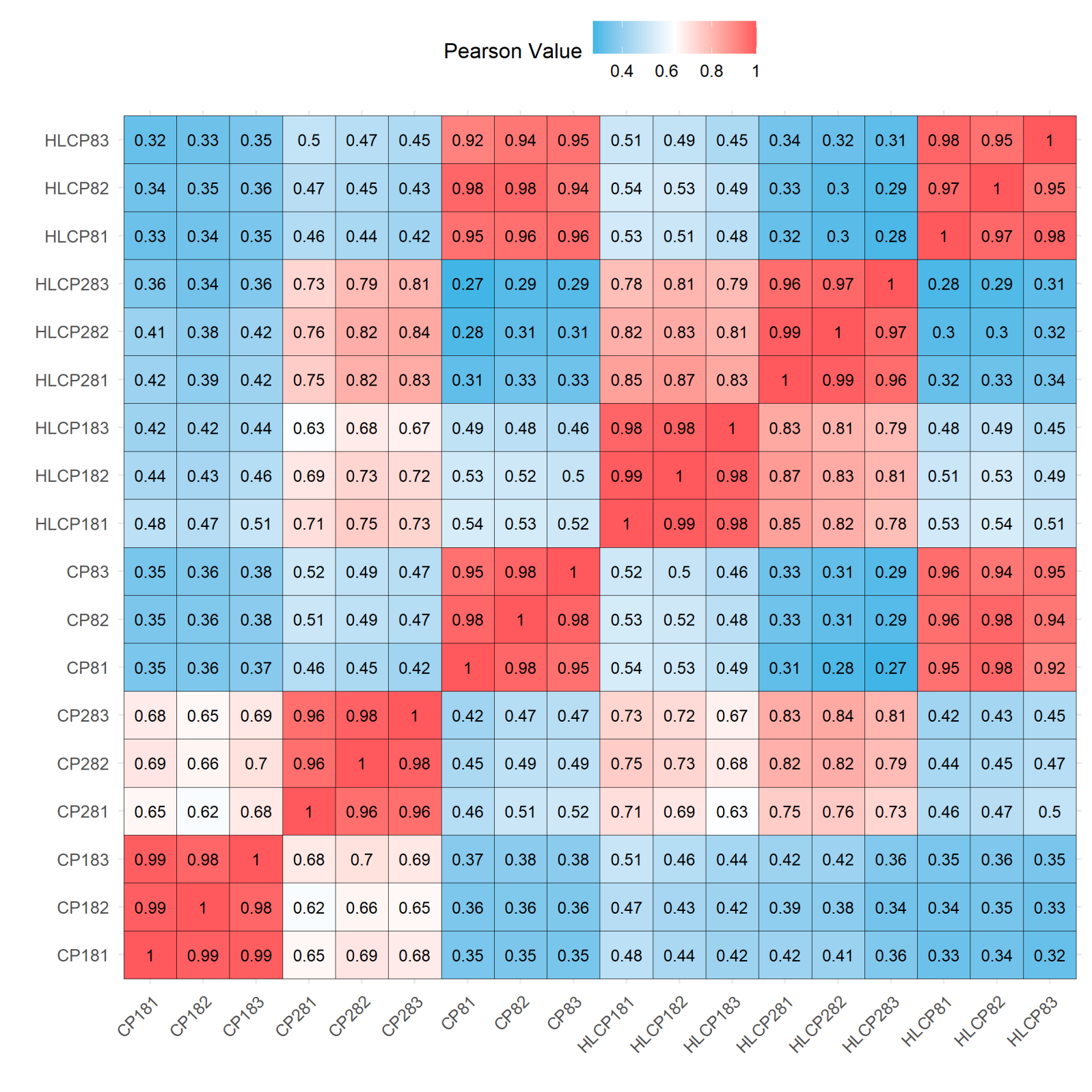
**Figure S3.** Correlation analysis of 18 samples in the seed coat of hulled and hull-less *C. pepo*.


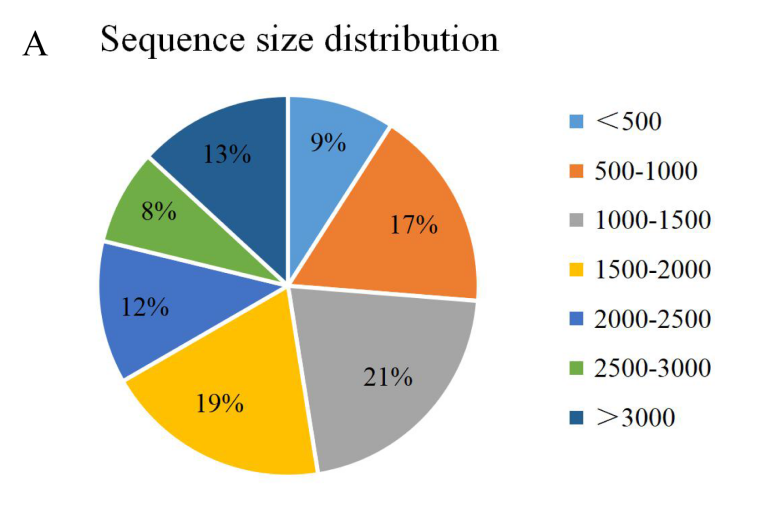

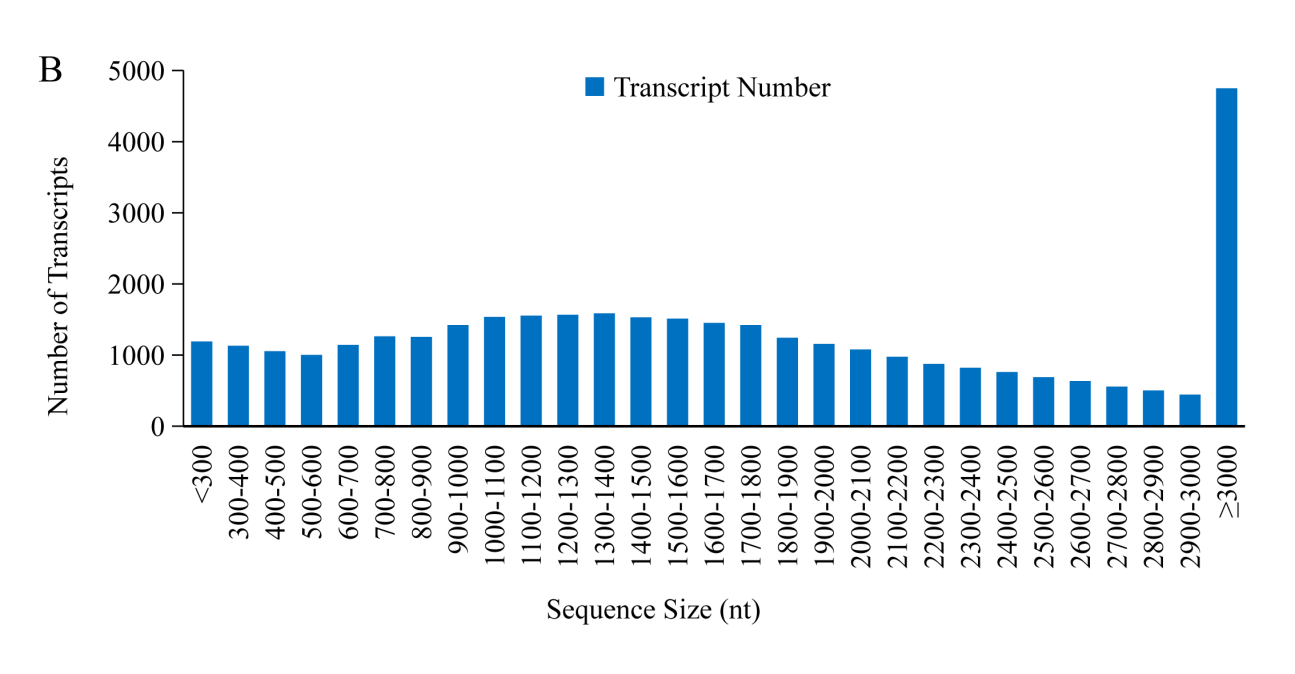


**Figure S4.** Length distribution figure (A, B) of RNA-Seq.


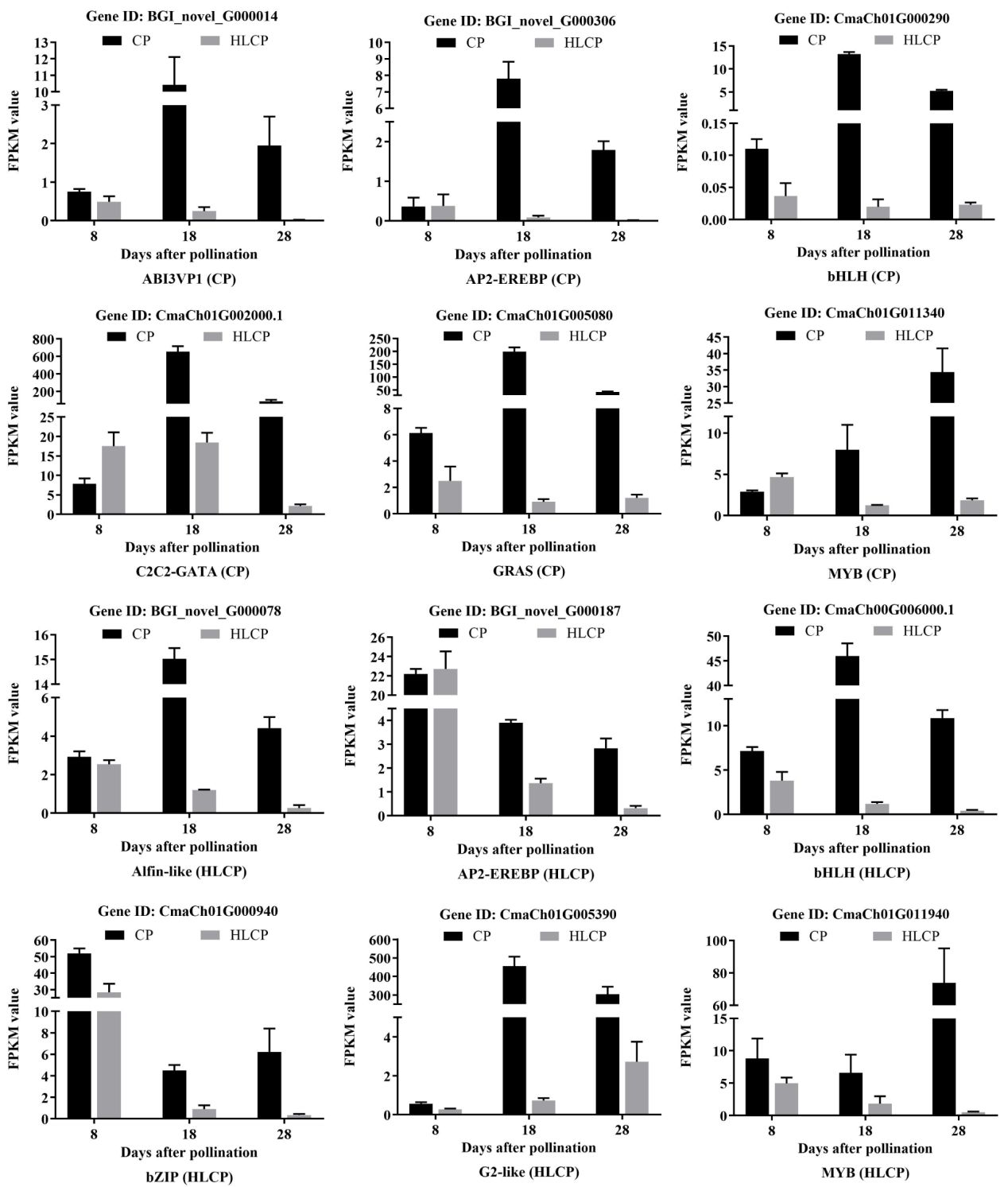


**Figure S5.** FPKM values of genes in transcription factors of hulled and hull-less *C. pepo*. Among the 12 transcription factors, 6 genes were selected from the transcription factors of hulled *C. pepo*, the other 6 genes were selected from the transcription factors of hull-less *C. pepo*. The black bar indicates the FPKM values of hulled *C. pepo*. The FPKM values of hull-less *C. pepo*. is represented by means of a gray histogram. Error bars represent standard error calculated from three replicates.
